# RFWD3 Reprograms Nucleotide Metabolism Through PHGDH to Induce Chemoresistance In Osteosarcoma

**DOI:** 10.1002/advs.202410937

**Published:** 2025-02-28

**Authors:** Wenchao Zhang, Chi Yin, Lin Qi, Zhongyue Liu, Ruiling Xu, Chao Tu, Zhihong Li

**Affiliations:** ^1^ Department of Orthopedics The Second Xiangya Hospital Central South University Changsha 410011 China; ^2^ Hunan Key Laboratory of Tumor Models and Individualized Medicine The Second Xiangya Hospital Changsha 410011 China; ^3^ Department of Neurosurgery The Second Xiangya Hospital Central South University Changsha 410011 China; ^4^ Changsha Medical University Changsha 410219 China; ^5^ Shenzhen Research Institute of Central South University Guangdong 518063 China; ^6^ FuRong Laboratory Changsha Hunan 410078 China

**Keywords:** chemoresistance, cisplatin, nucleotide metabolism, osteosarcoma, RFWD3

## Abstract

Chemoresistance represents a major challenge for osteosarcoma treatment. Despite the improved knowledge of cancer biology, the core determinants of cisplatin (DDP) resistance in osteosarcoma remain unclear and deserve further exploration. Here, RFWD3 is identified as a key regulator of DDP sensitivity in osteosarcoma using a genome‐wide CRISPR screen. It is demonstrated that RFWD3 is overexpressed in post‐chemotherapy osteosarcoma tissues compared to pre‐chemotherapy tissues. Knocking out RFWD3 increased the sensitivity of osteosarcoma cells to DDP treatment. Mechanistically, RFWD3 bound to and ubiquitinated PHGDH at the Lys137 residue, promoting its degradation and conserving cellular oxidized nicotinamide adenine dinucleotide (NAD^+^). The resulting surplus of NAD^+^ enhanced the TCA cycle, leading to increased production of aspartic acid and glutamic acid for de novo nucleotide biosynthesis. In addition, virtual screening techniques are employed to identify Lomitapide as a specific inhibitor of the RFWD3‐PHGDH interaction, capable of disrupting the binding between RFWD3 and PHGDH. It is found that Lomitapide exhibits a significant synergistic anti‐osteosarcoma effect when combined with DDP. In conclusion, a specific role of RFWD3 in regulating nucleotide metabolism is revealed and comprised of targetable candidates for overcoming chemoresistance in osteosarcoma.

## Graphical abstract

RFWD3 is identified as a key regulator of cisplatin (DDP) sensitivity in osteosarcoma using a genome‐wide CRISPR screen. RFWD3 promotes PHGDH ubiquitination, enhancing NAD+‐mediated nucleotide biosynthesis. Knocking out RFWD3 sensitized osteosarcoma cells to DDP. Additionally, Lomitapide, a specific RFWD3‐PHGDH interaction inhibitor, synergistically enhanced the anti‐osteosarcoma effect of DDP, offering potential therapeutic insights.

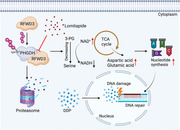

## 1 Introduction

Osteosarcoma is the most prevalent primary bone malignancy, with an annual incidence of ≈1–3 cases per million.^[^ ^1^ ^]^ The standard treatment for osteosarcoma currently includes surgery and chemotherapy. However, the core components of chemotherapy (cisplatin (DDP), doxorubicin, methotrexate, and ifosfamide) have remained unchanged for over 30 years. Some patients experience poor prognosis due to the development of drug resistance.^[^ ^2^ ^]^ Therefore, elucidating the underlying mechanisms of chemoresistance is urgently needed.

Augmentation of nucleotide metabolism is a key hallmark of cancer cells, contributing to many aspects of cancer cell behaviors, such as metastasis and therapy resistance.^[^ ^3^ ^]^ For instance, alteration in nucleotide biosynthesis fuels 5‐Fluorouracil resistance in a serine hydroxymethyltransferase‐2 dependent manner.^[^ ^4^ ^]^ CSN6 promotes the development and chemoresistance of colorectal cancer through upregulating purine and pyrimidine biosynthesis.^[^ ^5^ ^]^ Glutamine Synthetase facilitates radiation resistance of cancers by enhancing nucleotide metabolism, which subsequently accelerates DNA damage repair.^[^ ^6^ ^]^ Cisplatin induces DNA damage by covalently binding to the N7 position of purine bases, forming severe intra‐strand crosslinks.^[^ ^7^ ^]^ In response to cisplatin, cells activate various DNA repair pathways. Adequate nucleotide pools, as the raw materials for DNA synthesis, are essential for effective DNA repair. Therefore, targeting nucleoside metabolism is an efficient strategy to overcome cisplatin resistance.^[^ ^8^ ^]^ However, the underlying mechanisms and potential strategies based on nucleotide metabolism remain unexplored in osteosarcoma.

Ring Finger and WD Repeat Domain 3 (RFWD3) is a RING‐type E3 ligase. Previous studies have reported that RFWD3 interacted with RPA and regulated its ubiquitination in DNA‐damage‐induced foci to mediate homologous recombination.^[^ ^9^ ^]^ Also, RFWD3 promotes the replication forks by enhancing the recruitment of DNA translocase ZRANB3 to damage sites.^[^ ^10^ ^]^ Ubiquitination of proteins on single‐stranded DNA by RFWD3 is essential for responding to single‐stranded DNA gap.^[^ ^11^ ^]^ Despite this, the role of RFWD3 in drug resistance remains largely unexplored. Phosphoglycerate dehydrogenase (PHGDH) is a rate‐limiting enzyme of serine synthesis that catalyzes 3‐phosphoglycerate to produce 3‐phospohydroxy‐pyruvate by consuming NAD^+^. Activation of PHGDH contributes to tumor growth, tumor metastasis, and chemoresistance.^[^ ^5^, ^12^, ^13^ ^]^ However, the role of PHGDH in the chemoresistance of osteosarcoma remains unclear.

In the current study, we utilized a genome‐wide CRISPR screen to identify key drivers of cisplatin resistance in osteosarcoma. The results showed that RFWD3 knockout significantly reduced chemoresistance in osteosarcoma. Further investigation revealed that, in addition to regulating DNA repair proteins, RFWD3 reprograms nucleotide metabolism in osteosarcoma cells to induce cisplatin resistance. These findings offer a new perspective on treating osteosarcoma, suggesting that targeting RFWD3‐mediated metabolism pathways may be a promising strategy to overcome chemoresistance.

## 2 Experimental Section

### 2.1 Clinical Samples

Osteosarcoma samples were collected from the Department of Orthopedics at The Second Xiangya Hospital, Central South University. Written informed consent was obtained from all patients. The ethics committee of The Second Xiangya Hospital approved the use of these osteosarcoma tissues (Approval no. Z0859‐01). Specimens were gathered both before and after neoadjuvant chemotherapy for subsequent analysis.

### 2.2 Cell Lines and Cell Culture

The osteosarcoma cell lines (143B and U2OS) and HEK293T were purchased from Procell Life Science&Technology Co., Ltd (Wuhan, China). 143B and HEK293T cells were cultured in DMEM (Procell, China) supplemented with 10% fetal bovine serum (Newzurm, China) and 1% Penicillin–Streptomycin (NCM biotech, China). U2OS cells were cultured in McCoy's 5A medium, completed with 10% fetal bovine serum and 1% Penicillin–Streptomycin. A TransDetect PCR Mycoplasma Detection Kit (TransGen Biotech, China) was used to detect mycoplasma contamination every 3 months.

### 2.3 Quantitative Real‐Time PCR (RT‐qPCR)

Total RNA was extracted from osteosarcoma cells using the TRIzol reagent (Thermo Fisher Scientific) following the manufacturer's protocol. RNA quantity and purity were assessed with a NanoDrop 2000 spectrophotometer (Thermo Fisher Scientific). Reverse transcription was carried out using Hifair III Reverse Transcriptase (Yeasen Biotechnology Co., Ltd., China) to generate cDNA. Subsequent quantitative real‐time PCR (RT‐qPCR) was performed on a QuantStudio 5 system (Applied Biosystems, USA) utilizing SYBR Green Master Mix (Accurate Biology, China, #AG11746). Gene expression levels were normalized against GAPDH and quantified using the 2–ΔCt method. The sequences for all primers used are listed in Table S1 (Supporting Information).

### 2.4 Western Blotting (WB)

After the indicated treatment, cells were lysed using radioimmunoprecipitation assay (RIPA) buffer (#P0013, Beyotime). Protein concentrations were measured using the Micro BCA Protein Assay Kit (Sigma–Aldrich). The protein lysates were separated on SDS‐PAGE gels and transferred onto PVDF membranes. The membranes were blocked with 5% skim milk at room temperature for one hour. Subsequently, the proteins were incubated with specific primary antibodies overnight at 4 °C. After washing three times with TBST, the membranes were incubated with secondary antibodies for one hour at room temperature. Enhanced chemiluminescence (ECL) reagent was then added dropwise onto the PVDF membranes to detect protein expression. The antibodies used were as follows: RFWD3 (#H00055159‐M01, abnova, 1:500 dilution); PHGDH (#67591‐1‐Ig, proteintech, 1:5000 dilution); Flag (#20543‐1‐AP, proteintech, 1:5000 dilution); Myc(#16286‐1‐AP, proteintech, 1:5000 dilution); HA (#51064‐2‐AP, proteintech, 1:5000 dilution); beta‐Actin (#66009‐1‐Ig, proteintech, 1:5000 dilution); ENO1 (#sc‐390163, santa cruz, 1:500 dilution); Tubulin (#66240‐1‐Ig, proteintech, 1:5000 dilution). Vinculin (#66305‐2‐Ig, proteintech, 1:5000 dilution).

### 2.5 Colony Formation Assay

Osteosarcoma cells were seeded into 6‐well plates at a density of 500–1000 cells per well. The next day, the cells were treated with the indicated drugs and cultured for two weeks to allow colony formation, with the medium being replaced every three days. Finally, the cells were fixed with 4% paraformaldehyde for 30 min and stained with crystal violet.

### 2.6 CCK‐8 Assay

Osteosarcoma cells were seeded into 96‐well plates at a density of 2000 cells per well. After 24 h, the cells were treated with the appropriate drugs and incubated for the specified durations. At the end of the treatment period, 10 µL of sterile CCK‐8 solution was added to each well, followed by incubation at 37 °C for one hour. The absorbance at 450 nm was then measured.

### 2.7 Transfection of siRNA and Plasmids

Cells were seeded into suitable plates and cultured overnight to prepare for transfection. Plasmids/siRNAs and Lipofectamine 2000 (Thermo Fisher Scientific) were each incubated separately with serum‐free Opti‐MEM medium for 5 min. Afterward, the plasmid/siRNA and Lipofectamine mixtures were combined and incubated for 15 min. The resulting mixtures were added to the cell plates, and transfection was carried out for 6 h.

### 2.8 Lentivirus Construction and Transduction

HEK293T cells were plated at 2–3 × 10^^6^ cells per 10 cm dish and incubated overnight at 37 °C. The cells were transfected with 12 µg lentiviral vector, 6 µg psPAX2, and 6 µg pMD2.G using Lipofectamine 2000. The supernatant was collected after 48 and 72 h, centrifuged, and filtered. For transduction, 2 µg mL^−1^ Polybrene was added to the viral supernatant, the medium on osteosarcoma cells at 50%–60% confluency was replaced, and the cells were incubated for 24 h. The medium was replaced with fresh medium, and puromycin selection was initiated 48–72 h later.

### 2.9 Metabolomics Analysis

The metabolomic data analysis was performed by Shanghai Luming Biological Technology Co., LTD (Shanghai, China) using an ACQUITY UPLC I‐Class Plus (Waters Corporation) with a Q‐Exactive mass spectrometer and a heated ESI source (Thermo Fisher Scientific). Metabolic profiling in both ESI positive and negative ion modes employed an ACQUITY UPLC HSS T3 column (1.8 µm, 2.1 × 100 mm). The gradient elution used water and acetonitrile with 0.1% formic acid. The flow rate was 0.35 mL min^−1^ at 45 °C. Samples were kept at 10 °C, and the injection volume was 5 µL. The mass range was m/z 100 to 1000, with resolutions of 70000 for full MS scans and 17500 for HCD MS/MS scans. Collision energy was set at 10, 20, and 40 eV. The mass spectrometer settings were: spray voltage 3800 V (+) and 3200 V (−), sheath gas flow rate 35 units, auxiliary gas flow rate 8 units, capillary temperature 320 °C, auxiliary gas heater temperature 350 °C, S‐lens RF level 50.

### 2.10 Metabolites Detection

The intracellular metabolites were detected using corresponding detection kits according to their manufacturer's protocol, including the Aspartate Colorimetric Assay Kit (Elabscience, E‐BC‐K849), Glutamic Acid Colorimetric Assay Kit (Elabscience, E‐BC‐K118), Human Serine Assay Elisa Kit (COIBO BIO, CB11313‐Hu), Human Glycine Assay Elisa Kit (COIBO BIO, CB13081‐Hu) and NAD^+^/NADH Assay Kit (Beyotime, S0175).

### 2.11 Genome‐Wide CRISPR/Cas9 Knockout Library Screen

**Figure 1:**
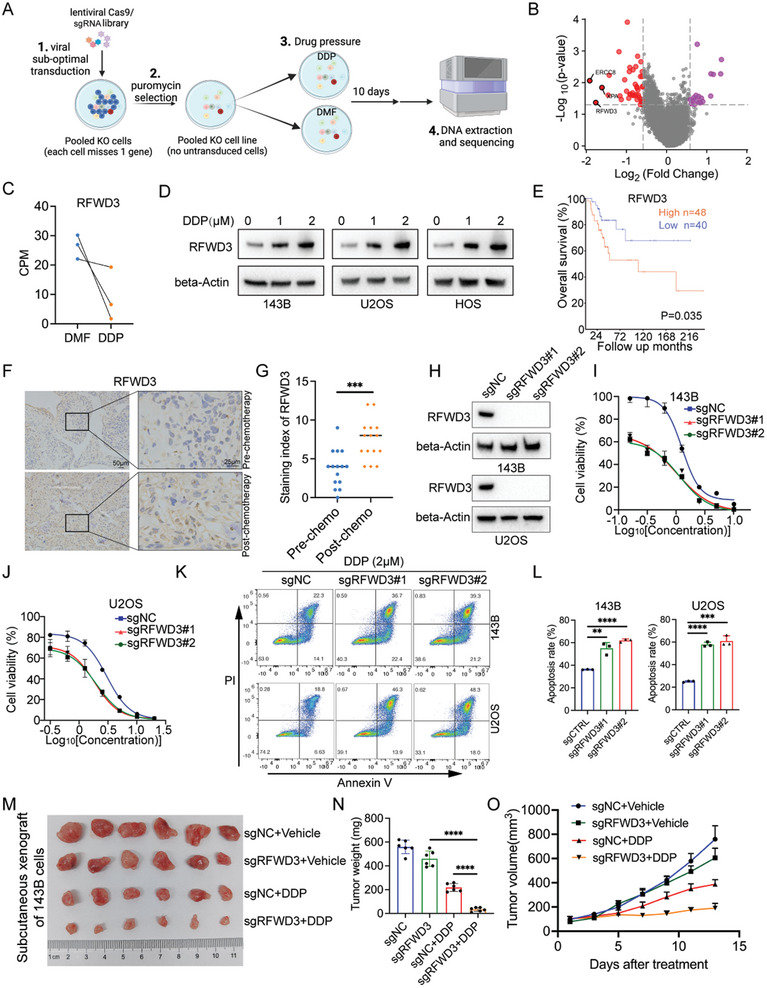
CRISPR screen identifies RFWD3 as a key regulator of DDP resistance in osteosarcoma. A) Workflow of the genome‐wide CRISPR screen in osteosarcoma cells. B) Volcano plot showing the enriched targeting genes of the screen. C) Comparisons of the CPM values of the top negatively enriched targeting genes between the DDP‐ and DMF‐treated groups. D) 143B, HOS, and U2OS cells were treated with the indicated concentration of DDP for 48h, and the WCLs were harvested for western blot analysis. E) Kaplan‐Meier analysis of patients with high and low RFWD3 expression using the R2 database. F) IHC staining of RFWD3 in osteosarcoma tissues from patients pre‐ and post‐ chemotherapy. G) Quantification of the IHC score in the indicated comparisons. Data are presented as the mean ± SD with three replicates. ^***^ *P* < 0.001. H) 143B and U2OS cells were transfected with indicated constructs for 48 h. After puromycin selection, these cells were subjected to Western blotting analysis. I–L) 143B and U2OS cells were transfected with indicated constructs for 48 h. After puromycin selection, these cells were subjected to CCK‐8 assay and Annexin V‐FITC/PI assay with the indicated treatment. sgNC, cells transfected with the non‐targeting sgRNA, sgRFWD3, cells transfected with the sgRNA targeting RFWD3. **P < 0.01, ***P < 0.001, ****P < 0.0001. M–O) 143B cells were transfected with the indicated constructs for 48 h. After puromycin selection, these cells were subcutaneously injected into the nude mice before treating the mice with or without DDP (5 mg kg^−1^, twice a week, intraperitoneally). Tumor growth curve (M), tumor images (N), and tumor mass (O) are shown. Data are presented as the mean ± SD with six replicates. ^****^ *P* < 0.0001.

The human Genome‐wide CRISPR/Cas9 library was a gift from Feng Zhang (Addgene # 1000000048).^[^ ^14^ ^]^ The flowchart of the screen is illustrated in **Figure** **1**A. First, 143B cells were transfected with the genome‐wide CRISPR/Cas9 library (containing 65 384 sgRNAs targeting 19 032 human genes, 1 822 miRNAs, and 1 000 non‐targeting controls) at a low MOI of 0.3 and a coverage of 500X. The transduced cells were then selected with 2 µg mL^−1^ puromycin for seven days. Subsequently, at least 3 × 10^7^ mutant cells were treated with DDP or DMF for 10 days respectively. At the end of treatment, ≈3 × 10^7^ cells in each group were collected for genomic DNA extraction. The sgRNA sequences were amplified using NEBNext High‐Fidelity 2X PCR Master Mix. The products were then purified and sequenced by Genergy Biotechnology (Shanghai, China). Data analysis was performed using the MAGeCK v0.5.7 algorithm.^[^ ^15^ ^]^

### 2.12 Immunohistochemistry (IHC)

Paraffin‐embedded clinical samples were used for IHC analysis as previously reported^2^. The following antibodies were used for IHC staining: RFWD3 (Proteintech, 19893‐1‐AP) and PHGDH (Santa Cruz, sc‐390610). Two independent pathologists reviewed the slice and calculated the IHC index by multiplying the density score (0/1/2/3) and positive rate score (1/2/3/4).

### 2.13 Flow Cytometry

The apoptosis rate was measured using the Annexin V‐FITC/PI assay per the manufacturer's instructions. Cells were stained with Annexin V‐FITC and Propidium Iodide (PI) from the Annexin V‐PE/PI Apoptosis Detection Kit (#40302ES60, YEASEN) for 10 min, then detected with a Cytek NL‐CLC. Data analysis was performed using FlowJo software (FlowJo, USA).

### 2.14 Comet Assay

First, cells were digested and mixed with low‐melting‐point agarose. This mixture was then spread onto microscope slides pre‐coated with a thin agarose layer. The cells were lysed and exposed to electrophoresis in an alkaline buffer. After neutralizing, the DNA was stained with Propidium Iodide (PI) (#C2041S‐5, Beyotime) and examined using a fluorescence microscope to visualize the DNA fragments.

### 2.15 Co‐Immunoprecipitation (Co‐IP) and Liquid Chromatography Coupled to Tandem Mass Spectrometry (LC‐MS/MS)

Proteins were extracted using IP buffer and incubated for 24 h with Anti‐Myc Affinity Gel (#P2285, Beyotime), Anti‐Flag Affinity Gel (#P2071, Beyotime), or Protein A+G Magnetic Beads (#P2108, Beyotime) conjugated with the primary antibody/IgG. The beads were then collected and washed three times with PBS. The bound proteins were eluted by boiling with an SDS‐loading buffer for 10 min. Subsequently, the eluted proteins were subjected to gel electrophoresis and analyzed via LC‐MS/MS. The antibodies used were as follows: Rabbit polyclonal to RFWD3 (#ab99306, abcam), Rabbit polyclonal to PHGDH (#ab240744, abcam), IgG (#A7016, Beyotime).

### 2.16 Molecular Docking

Rigid protein‐protein docking between RFWD3 and PHGDH was performed using GRAMM‐X to investigate their interactions. Protein structures were sourced from the UniProt database (www.uniprot.org), the PDB database (RCSB PDB: Homepage), and the AlphaFold database. PyMOL (Version 2.4) was utilized for the analysis of protein interactions and subsequent visualization.

### 2.17 Virtual Screening

Molecular docking studies were performed using the semi‐flexible docking algorithm of AutoDock Vina (version 1.1.2) and were compiled and run under the Windows 10 operating system. The crystal structure of RFWD3 was defined as a receptor. The 2115 compounds from the ZINC database were defined as the virtual screening library. All compounds were prepared using OpenBabel. The docking poses were scored using the Vina scoring function, which considers various intermolecular interactions like van der Waals forces and hydrogen bonds. Up to 10 conformations of each ligand were considered in the docking process, and we selected the top five compounds with the lowest binding energies (≤ −7 kcal mol^−1^) as candidates for further molecular dynamics analysis via GROMACS. All structural reconstructions were produced using Pymol.

### 2.18 Drug Affinity Responsive Target Stability

Cells were harvested, and total proteins were extracted using lysis buffers. The cell lysates were centrifuged at 12000 g for 15 min at 4 °C. The resulting supernatant was treated with either DMSO or lomitapide (1 and 10 µm). After incubation at room temperature for 4 h, pronase (2 µg mL^−1^, Roche) was added to the lysates at room temperature and incubated for an additional 2 h. The reaction was terminated by the addition of a protease inhibitor cocktail and SDS‐PAGE sample buffer. At the end, samples were loaded onto an SDS‐PAGE gel and subjected to silver staining or Western blot analysis.

### 2.19 Cell Thermal Shift Assay

Approximately 1 × 106 cells were collected and washed with PBS, followed by lysis with NP‐40 lysis buffers (containing protease and phosphatase inhibitors) for 30 min at 4 °C. The samples were centrifuged at 12 000g for 10 min at 4 °C. Subsequently, the protein supernatant was treated with DMSO or lomitapide (10 µm). After incubation at room temperature for 1 h, the cells in each group were heated at the indicated temperature for 5 min and then centrifuged at 12000 g for 15 min at 4 °C. The supernatants were then processed for Western blotting.

### 2.20 Mouse Xenograft Assay

BALB/c nude mice were purchased from Hunan SJA Laboratory Animal Co., Ltd. (Hunan, China) and housed under pathogen‐free conditions. 5 × 10^6^ cells were subcutaneously injected into the right posterior flank of BALB/c nude mice (5 weeks old). Mice were randomly divided into groups with indicated treatment once the tumor volume reached 100 mm^3^. Tumor volume was measured every other day and calculated using the formula of 1/2 × length × width^2^. At the endpoint, the tumors were excised and weighed. The Institutional Animal Care and Use Committee of the Second Xiangya Hospital, Central South University, approved all the animal experiments (Approval No. 20230301).

### 2.21 Statistical Analysis

Statistical analysis was performed using GraphPad Prism 9. Data are expressed as mean ± SD. Group comparisons were made using an unpaired two‐sided Student's t‐test or one‐way ANOVA. A *P*‐value of less than 0.05 was considered statistically significant.

## 3 Results

### 3.1 CRISPR Screen Identifies RFWD3 as a Key Regulator of DDP Resistance in Osteosarcoma

To elucidate the primary determinants of DDP sensitivity in osteosarcoma, we conducted a comprehensive genome‐wide CRISPR screen (Figure 1A). We postulated that the knockout of genes conferring DDP resistance could potentiate the antitumor efficacy of DDP. Our analysis identified 75 genes that were significantly negatively enriched in the DDP‐treated group (log2FC > 0.5, *P* < 0.05) compared to the DMF group (Figure 1B). Among these, the top three enriched genes were ERCC8, RFWD3, and XPA. Notably, ERCC8 and XPA have been previously implicated in DDP resistance,^[^ ^16^, ^17^ ^]^ and we also demonstrated that they regulated the sensitivity of osteosarcoma cells to DDP treatment (Figure S1A,B, Supporting Information). Consequently, we focused our investigation on the role of RFWD3 in mediating chemoresistance (Figure 1C). We subsequently examined the expression profile of RFWD3 in response to DDP treatment. The results demonstrated a significant upregulation of RFWD3 upon DDP exposure, suggesting that RFWD3 is a DDP‐responsive protein (Figure 1D). Further survival analysis utilizing the R2 online database revealed that elevated RFWD3 expression correlates with poor prognosis in osteosarcoma patients (Figure 1E). Additionally, we assessed RFWD3 expression levels in osteosarcoma tissues pre‐ and post‐chemotherapy. Our findings indicated a marked increase in RFWD3 expression in post‐chemotherapy specimens (Figure 1F,G). Collectively, these results imply that RFWD3 is intricately linked to DDP treatment response.

To further validate the impact of RFWD3 on DDP resistance in osteosarcoma, we generated RFWD3 knockout cells utilizing the CRISPR/Cas9 system. The successful knockout of RFWD3 was verified via Western blot analysis in 143B and U2OS cell lines (Figure 1H). Our results demonstrated that the ablation of RFWD3 significantly impeded cell proliferation and induced apoptosis upon DDP treatment (Figure 1I–L; Figure S1C,D, Supporting Information). Furthermore, we wondered whether RFWD3 specifically regulates the sensitivity to DDP only in osteosarcoma cells. To address this, we first examined the expression of RFWD3 in osteosarcoma cells and three other cell types from the tumor microenvironment (hMBSC, hFOB1.19, and HSF). We found that RFWD3 expression was significantly higher in osteosarcoma cells compared to the other three cell types (Figure S1E, Supporting Information). Meanwhile, we found that RFWD3 also regulates the sensitivity of other malignancies to DDP treatment, such as melanoma and breast cancer (Figure S1F, Supporting Information). This suggests that RFWD3 may play a role in developing drug resistance in various types of tumors.

Additionally, we assessed the effect of RFWD3 on DDP sensitivity in an in vivo mouse model. 143B cells, with and without RFWD3 knockout, were subcutaneously implanted into the right posterior flank of BALB/c nude mice. These mice were administered DDP or vehicle for 14 days. The data revealed that the loss of RFWD3 markedly enhanced the sensitivity of osteosarcoma to DDP treatment (Figure 1M–O). Collectively, these findings indicate that RFWD3 is a pivotal regulator of DDP sensitivity in osteosarcoma.

### 3.2 RFWD3 Interacts and Destabilizes PHGDH in a Proteasome‐Dependent Manner

**Figure 2:**
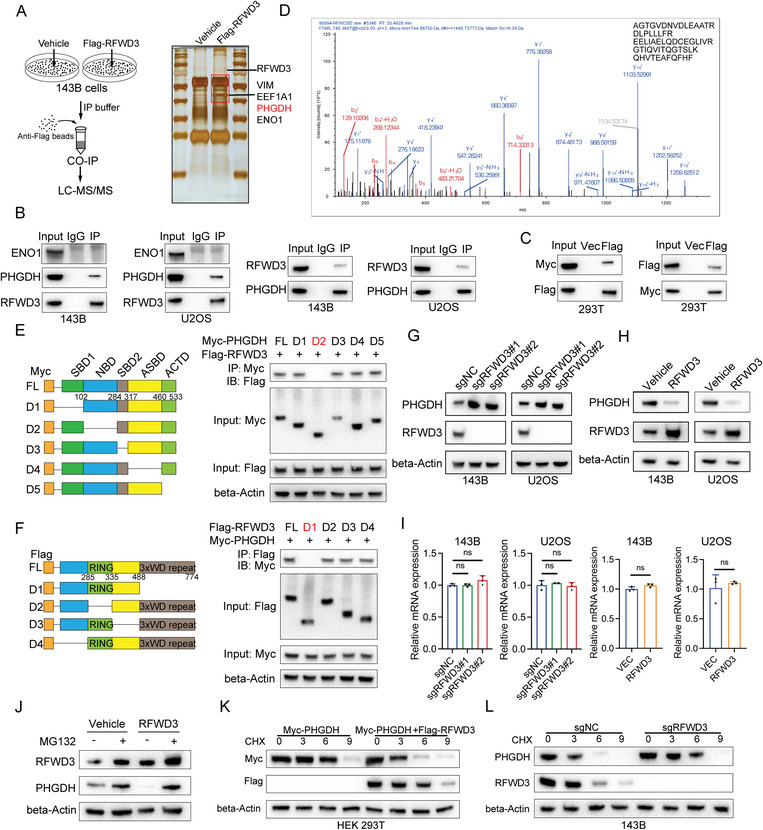
RFWD3 interacts and destabilizes PHGDH in a proteasome‐dependent manner. A) 143B cells were transfected with Flag‐RFWD3 or Vehicle, the whole cell lysate of 143B cells was harvested for immunoprecipitation (IP) assay using anti‐Flag antibody and subjected to silver staining and LC‐MS/MS analysis. B) The WCLs of 143B and U2OS cells were harvested for Co‐IP assay using anti‐RFWD3 and anti‐PHGDH antibodies. C) CO‐IP analysis of HEK293T cells transfected with Myc‐PHGDH and Flag‐RFWD3. D) PHGDH peptide fragment was precipitated with Flag‐RFWD3 by MS. E) Schematic representation of Myc‐PHGDH truncating mutants was shown (upper panel). HEK293T cells were cotransfected with Flag‐RFWD3 and indicated Myc‐PHGDH mutants. The cells were lysed for immunoprecipitation using anti‐Myc antibody, and anti‐Flag antibody was used to detect the protein binding (lower panel). F) A schematic representation of RFWD3 truncating mutants was shown (upper panel). The truncated mutants of Flag‐RFWD3 and full‐length Myc‐PHGDH were cotransfected in HEK293T cells, followed by analysis by CO‐IP. G,H) 143B and U2OS cells were transfected with indicated constructs for 48 h. After puromycin selection, these cells were subjected to Western blotting. I) 143B and U2OS cells were transfected with the indicated constructs for 48 h. After puromycin selection, the cells were subjected to qRT–PCR. Data are presented as the mean ± SD of three independent experiments. ns: Not significant. J) 143B and U2OS cells were transfected with the indicated constructs for 48 h. After puromycin selection, these cells were treated with or without 10µm MG132 for 6h and were then subjected to Western blotting. K) Myc‐PHGDH and Flag‐RFWD3/Vehicle were transfected into HEK293T cells and treated with 100 µg mL^−1^ CHX for indicted time gradient (0, 3, 6, 9h) and were analyzed by Western blotting. L) 143B cells were transfected with indicated constructs for 48h. After puromycin selection, these cells were treated with 100 µg mL^−1^ CHX for indicted time gradient (0, 3, 6, 9 h) and were analyzed by Western blotting.

To elucidate the underlying mechanism by which RFWD3 modulates the sensitivity of osteosarcoma to DDP, 143B cells were transfected with a Flag‐RFWD3 construct for immunoprecipitation (IP) and subsequent LC‐MS/MS analysis (**Figure** **2**A). We analyzed the top five interactors of RFWD3, including VIM, PLEC, EEF1A1, PHGDH, and ENO1. Notably, PHGDH and ENO1 have been previously implicated in platinum response.^[^ ^18^, ^19^ ^]^ Consistently, we also identified that PHGDH and ENO1, but not other potential regulators, modulate the sensitivity of osteosarcoma cells to DDP (Figure S2A, Supporting Information). Co‐immunoprecipitation analysis demonstrated that PHGDH, but not ENO1, specifically interacted with RFWD3 (Figure 2B–D). Domain mapping analysis revealed that the nucleotide‐binding domain (NBD, amino acids 103–284) of PHGDH is responsible for its interaction with RFWD3 (Figure 2E). Conversely, RFWD3 interacts with PHGDH through its amino acids 488–774 region (Figure 2F).

Subsequently, we examined the impact of RFWD3 on PHGDH expression. Knockout of RFWD3 led to a significant upregulation of PHGDH protein levels in both 143B and U2OS cells, while overexpression of RFWD3 resulted in a marked downregulation of PHGDH protein (Figure 2G,H). Notably, the mRNA levels of PHGDH remained unchanged upon modulation of RFWD3 expression (Figure 2I), suggesting that RFWD3 regulates PHGDH at the post‐translational level. Given that RFWD3 functions as an E3 ubiquitin ligase, we investigated whether its influence on PHGDH is mediated through ubiquitination‐dependent proteasomal degradation. Treatment with the proteasome inhibitor MG132 abrogated the RFWD3‐induced reduction of PHGDH protein levels (Figure 2J). Subsequently, the degradation speed of PHGDH was detected while cells were treated with a protein synthesis inhibitor, cycloheximide (CHX). Results showed that ectopic expression of RFWD3 significantly decreased the half‐life of PHGDH, while deletion of RFWD3 stabilized endogenous PHGDH (Figure 2K,L; Figure S2B, Supporting Information). These results indicated that RFWD3 promotes the proteasomal degradation of PHGDH.

### 3.3 RFWD3 Ubiquitinates PHGDH on Lys137 in Response to DDP Treatment

**Figure 3:**
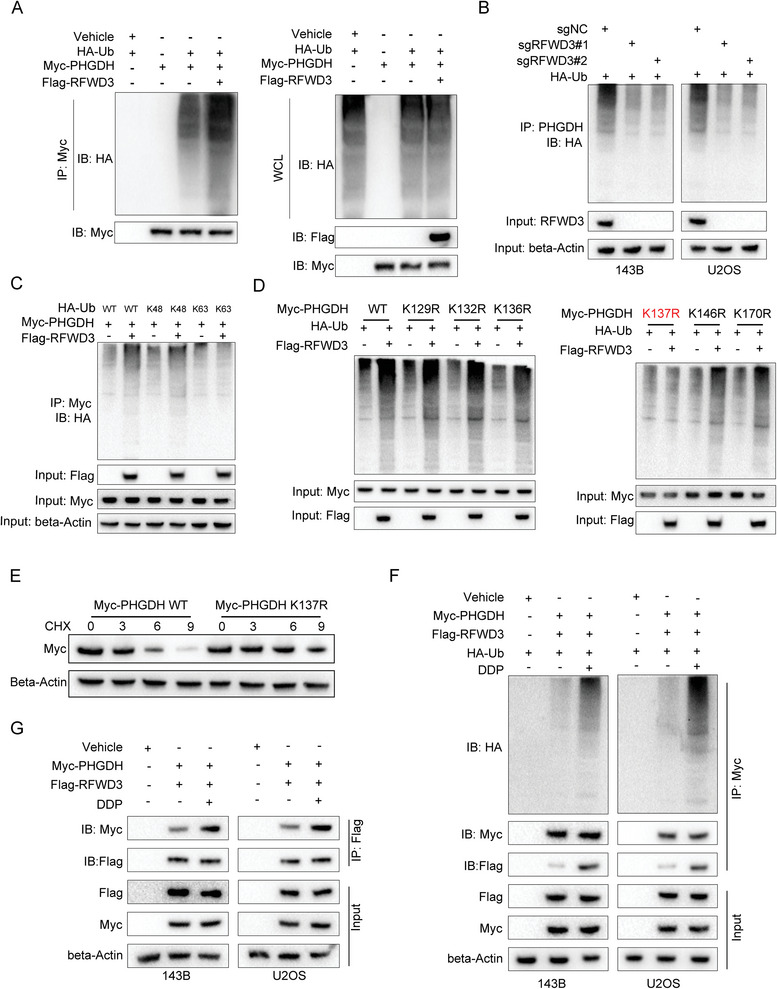
RFWD3 ubiquitinates PHGDH on Lys137 in response to DDP treatment. A) HEK293T cells were transfected with the indicated constructs. After 48 h of transfection, cells were treated with 10 µM MG132 for 6 h. Lysates were subjected to IP assays, followed by immunoblotting analysis. B) 143B and U2OS cells were transfected with indicated constructs for 48 h. After puromycin selection, cells were treated with 10 µM MG132 for 6 h. Lysates were subjected to IP assays, followed by immunoblotting analysis. C) HEK293FT cells were transfected with HA‐K48‐Ub/HA‐K63‐Ub/HA‐Ub and Myc‐PHGDH with or without Flag‐RFWD3 as indicated and cultured for 48 h. Cells were immunoprecipitated with anti‐Flag and analyzed. D) HEK293FT cells were transfected with HA‐Ub and different mutants of Myc‐PHGDH with or without Flag‐RFWD3 as indicated and cultured for 48 h. Cells were immunoprecipitated with anti‐Flag and analyzed. E) HEK293FT cells were transfected with WT Myc‐PHGDH or K137R Myc‐PHGDH and cultured for 48h, these cells were treated with 100 µg mL^−1^ CHX for indicted time gradient (0, 3, 6, 9 h) and were analyzed by Western blotting. F,G) 143B and U2OS cells were transfected with indicated constructs for 48 h and treated with or without DDP for 48 h. Cell lysates were subjected to IP assays, followed by immunoblotting analysis.

To elucidate the ubiquitination of PHGDH mediated by RFWD3, we performed a ubiquitination assay using HEK293T cells transfected with HA‐Ub, myc‐PHGDH, and flag‐RFWD3 constructs. Our results demonstrated that ectopic expression of RFWD3 significantly enhanced the polyubiquitination of PHGDH, while knockout of RFWD3 reduced the ubiquitination levels in 143B and U2OS cells (**Figure** **3**A,B). Ubiquitination involving lysine 48 (K48)‐ and K63‐linked polyubiquitin chains is typically associated with proteasomal degradation.^[^ ^20^ ^]^ To further dissect this, we co‐transfected myc‐PHGDH with HA‐Ub (wild type, K48‐only, or K63‐only) in the absence or presence of Flag‐RFWD3. Our findings revealed that RFWD3 markedly increased the K48‐linked ubiquitination of PHGDH without affecting K63‐linked ubiquitination (Figure 3C).

The former result revealed that RFWD3 is bound to the NBD domain of PHGDH, thus we supposed that lysine residues in this region may be responsible for ubiquitination by RFWD3. We identified seven lysine sites in this domain using the online ubiquitination prediction tool (https://cplm.biocuckoo.cn/). Subsequently, we generated PHGDH mutants with single lysine (K)‐to‐arginine (R) substitutions at each potential ubiquitination site for further analysis. Our findings indicate that the mutation of the K137 site abrogated the effect of RFWD3 on PHGDH ubiquitination (Figure 3D).

Additionally, the degradation rate of the K137R PHGDH mutant was significantly slower compared to the wild‐type PHGDH (Figure 3E). These results confirm that the K137 residue of PHGDH is essential for its ubiquitination by RFWD3. Furthermore, we investigated the impact of DDP on RFWD3‐mediated ubiquitination of PHGDH. Our results demonstrated that DDP treatment markedly augmented the interaction between RFWD3 and PHGDH (Figure 3F,G). Additionally, DDP treatment significantly elevated the ubiquitination levels of PHGDH (Figure 3F), indicating that PHGDH ubiquitination by RFWD3 may play a crucial role in osteosarcoma cells’ response to DDP treatment.

### 3.4 PHGDH is Partially Responsible for RFWD3‐Induced DDP Resistance in Osteosarcoma

**Figure 4:**
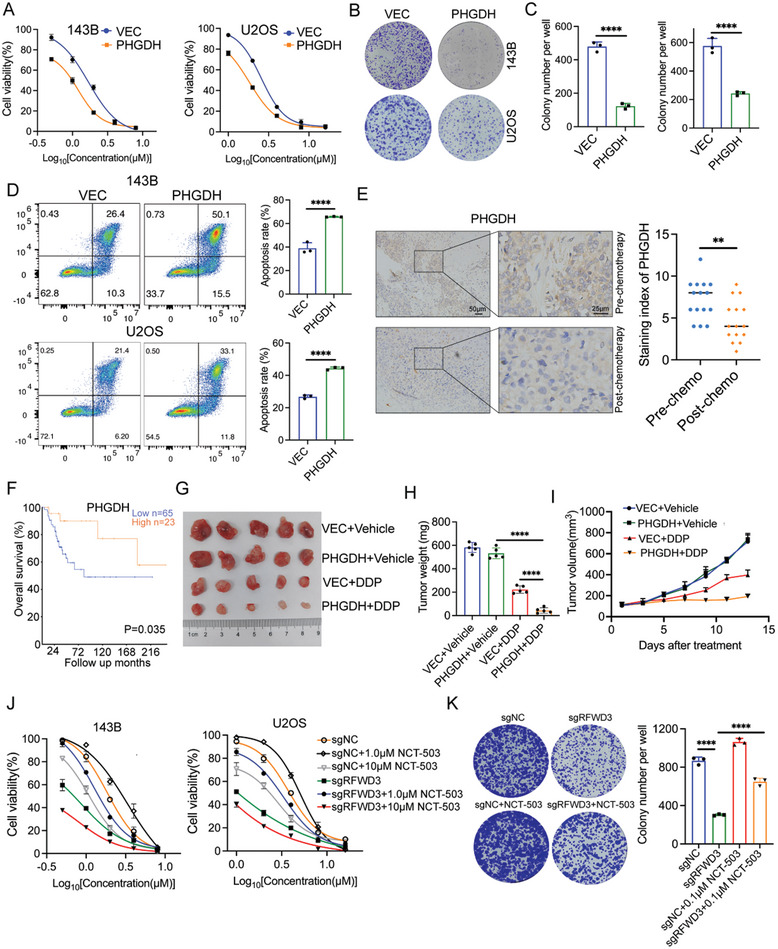
PHGDH is partially responsible for RFWD3‐induced DDP resistance in osteosarcoma. A–D) 143B and U2OS cells were transfected with indicated constructs for 48 h and treated with DDP for another 48 h, cells were subjected to CCK‐8 assay (A), colony formation assay (B,C), and Annexin V‐FITC/PI assay (D). Data are presented as the mean ± SD with three replicates. ^****^ *P* < 0.0001. E) IHC staining of PHGDH in osteosarcoma tissues from patients pre‐ and post‐ chemotherapy. **P < 0.01. F) Kaplan‐Meier analysis of patients with high and low PHGDH expression using the R2 database. G–I) 143B cells were transfected with the indicated constructs for 48 h. After puromycin selection, these cells were subcutaneously injected into the nude mice before treating the mice with or without DDP (5 mg kg^−1^, twice a week, intraperitoneally). Tumor growth curve (G), tumor images (H), and tumor mass (I) are shown. Data are presented as the mean ± SD with five replicates. ^****^ *P* < 0.0001. J,K) 143B and U2OS cells were transfected with indicated constructs for 48 h. After puromycin selection, these cells were treated with or without the indicated concentration of NCT‐503 for 24 h. These cells were then subjected to CCK‐8 assay (J) and colony formation assay (K). ****P < 0.0001.

Subsequently, we aimed to elucidate the role of PHGDH in DDP resistance in osteosarcoma. A PHGDH overexpression vector was transfected into 143B and U2OS cells. The results demonstrated that PHGDH overexpression markedly inhibited cell proliferation and induced apoptosis in the presence of DDP (**Figure** **4**A–D), while PHGDH inhibition increased the resistance of osteosarcoma cells to DDP (Figure S2C–G, Supporting Information). IHC analysis of clinical samples revealed that PHGDH expression was significantly reduced in post‐chemotherapy tissues compared to pre‐chemotherapy tissues (Figure 4E). Moreover, analysis of the R2 database indicated that low PHGDH expression was associated with poor patient prognosis (Figure 4F). Furthermore, in vivo experiments confirmed that PHGDH overexpression enhanced the sensitivity of osteosarcoma to DDP treatment (Figure 4G–I). Collectively, these findings underscore the pivotal role of PHGDH in DDP resistance in osteosarcoma.

To investigate whether RFWD3 mediates DDP resistance through the regulation of PHGDH, rescue experiments were conducted. The results showed that the effect of RFWD3 knockout on DDP sensitivity could be partially mitigated by a low dose of the PHGDH inhibitor NCT‐503 (Figure 4J,K), indicating that PHGDH partially mediated the RFWD3‐induced DDP resistance in osteosarcoma. However, we found that a high dose of NCT‐503 further inhibited the growth of RFWD3 knockout osteosarcoma cells under DDP treatment (Figure 4J). Previous studies have indicated that PHGDH activity is crucial for serine biosynthesis and tumor growth and that high doses of NCT‐503 reduce cell proliferation.^[^ ^13^ ^]^ The platinum‐resistant cells require exogenous serine for growth.^[^ ^19^ ^]^ A high dose of NCT‐503 may also disrupt the uptake of exogenous serine or suppress other metabolic processes in resistant cells, which deserves further investigation.

### 3.5 RFWD3 Increases Cellular NAD+ Level to Mediate DDP Resistance

**Figure 5:**
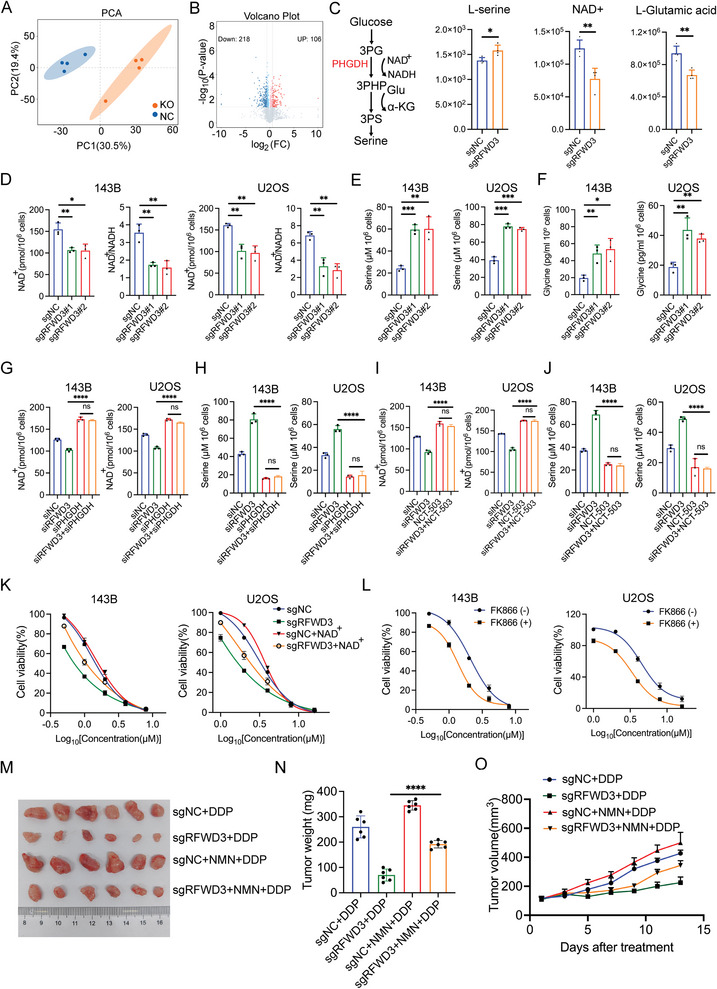
RFWD3 increases cellular NAD+ level to mediate DDP resistance. A) PCA analysis of the metabolomics data in RFWD3 knockout cells and relative control cells. B) The volcano plot showed the differential expressed metabolites between RFWD3 knockout cells and relative control cells. C) Level of metabolites related to PHGDH‐mediated serine synthesis in RFWD3 knockout cells and relative control cells. Data are presented as the mean ± SD with four replicates. ^*^ *P* < 0.05, ^**^ *P* < 0.01. D–F) 143B and U2OS cells were transfected with indicated constructs for 48 h. After puromycin selection, these cells were detected with NAD^+^ level and NAD^+^/NADH ratio (D), serine level (E), and glycine level (F). Data are presented as the mean ± SD with three replicates. ^*^ *P* < 0.05, ^**^ *P* < 0.01, ^***^ *P* < 0.001, ^****^ *P* < 0.0001. G,H) 143B and U2OS cells were transfected with indicated siRNAs for 48 h. These cells were then detected with NAD^+^ level (G) and serine level (H). Data are presented as the mean ± SD with three replicates. ns: Not significant, ^****^ *P* < 0.0001. I,J) 143B and U2OS cells were transfected with indicated siRNAs for 48 h and treated with or without NCT‐503 for 24 h. These cells were then detected with NAD^+^ level (I) and serine level (J). Data are presented as the mean ± SD with three replicates. Ns non‐significant, ^****^ *P* < 0.0001. K) 143B and U2OS cells were transfected with the indicated constructs for 48 h. After puromycin selection, these cells were treated with or without NAD^+^ for an additional 24 h under a range of DDP doses. Cell viability was assessed using the CCK‐8 assay. Data are presented as the mean ± SD from three replicates. L) 143B and U2OS cells were treated with or without FK866 under a range of DDP doses. Cell viability was assessed using the CCK‐8 assay. Data are presented as the mean ± SD from three replicates. M–O) 143B cells were transfected with the indicated constructs for 48 h. After puromycin selection, these cells were subcutaneously injected into the nude mice before treating the mice with or without NMN (300 mg kg^−1^, daily, intraperitoneally) under the treatment of DDP (5 mg kg^−1^, twice a week, intraperitoneally). Tumor growth curve (G), tumor images (H), and tumor mass (I) are shown. Data are presented as the mean ± SD with five replicates. ^****^ *P* < 0.0001.

Given the critical role of PHGDH as a metabolic enzyme, we proceeded to investigate the influence of RFWD3 on the metabolic pathways in osteosarcoma cells. Metabolomics analysis was performed on 143B cells transfected with either sgRFWD3 or sgNC. The results revealed distinct metabolic profiles between the sgRFWD3 and sgNC groups (**Figure** **5**A,B), in which 218 metabolites were significantly downregulated while 106 metabolites were upregulated (Fold change > |1.5|, *P*‐value < 0.05). We then integrated the alterations in metabolites associated with the PHGDH enzyme. Notably, RFWD3 knockout markedly elevated the levels of L‐serine, while decreasing the levels of NAD^+^ and L‐glutamic acid (Figure 5C). Further metabolite detection showed that RFWD3 knockdown reduced cellular NAD^+^ levels while increasing the levels of L‐serine and L‐glycine (Figure 5D–F). Next, the knockdown of PHGDH or inhibition of PHGDH activity could eliminate the effect of RFWD3 knockdown on NAD^+^ and serine level (Figure 5G–J), indicating that RFWD3 modulated osteosarcoma cell metabolism via PHGDH.

Additionally, DDP treatment was observed to raise NAD^+^ levels while reducing serine levels (Figure S3A–C, Supporting Information). However, although serine is involved in glycine synthesis, glycine levels did not change following cisplatin treatment. Glycine is an important precursor for nucleotide synthesis (Figure S3D, Supporting Information), indicating that osteosarcoma cells may have activated other pathways to produce glycine and maintain nucleotide biosynthesis. To further confirm that RFWD3 mediates DDP resistance by regulating cellular NAD+ level through PHGDH, we conducted in vivo rescue experiments. The results showed that RFWD3 knockout significantly increased PHGDH expression, reduced the NAD+/NADH ratio, and elevated serine levels of the subcutaneous tumor. PHGDH knockdown partially reversed the effects induced by RFWD3 knockout (Figure S3E–H, Supporting Information). To ascertain whether these metabolic changes were implicated in RFWD3‐mediated DDP resistance, RFWD3 knockout osteosarcoma cells were supplemented with NAD^+^ or serine. The results indicated that supplementation with NAD^+^, but not serine, could partially mitigate the effects of RFWD3 knockout on DDP resistance (Figure 5K; Figure S4A, Supporting Information). NAD^+^ is the major constraint for PHGDH activity, inhibition of PHGDH decreases the carbon flux through serine synthesis to spare NAD^+^.^[^ ^19^ ^]^ Moreover, the NAD^+^ synthesis inhibitor FK866 also increased the sensitivity of osteosarcoma cells to DDP (Figure 5L). Furthermore, the in vivo experiments confirmed that the administration of NAD^+^ precursor NMN could partially abolish the effect of RFWD3 knockout on DDP sensitivity (Figure 5M–O). Taken together, these findings confirmed that RFWD3 increased the cellular NAD^+^ level by regulating PHGDH to modulate DDP resistance.

### 3.6 RFWD3 Promotes Nucleotide Synthesis Through Increasing Aspartic Acid and Glutamic Acid Biogenesis

**Figure 6:**
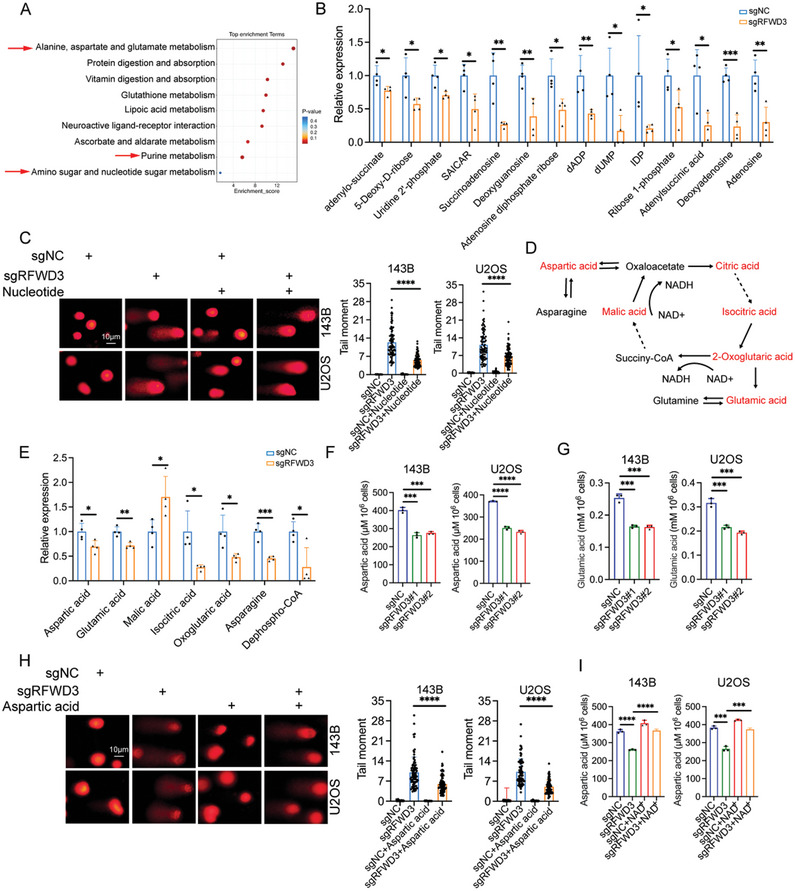
RFWD3 promotes nucleotide synthesis through increasing aspartic acid biogenesis. A) KEGG analysis of the differentially expressed metabolites between RFWD3 knockout cells and control cells. B) Level of metabolites of the pathways of nucleotide synthesis in RFWD3 knockout cells and control cells. Data are presented as the mean ± SD with four replicates. ^*^ *P* < 0.05, ^**^ *P* < 0.01, ^***^ *P* < 0.001. C) 143B and U2OS cells expressing sgNC or sgRFWD3 were treated with or without 12.5 µm purines (2′‐deoxyadenosine and 2′‐deoxyguanosine) and 12.5µm pyrimidines (2′‐deoxycytosine and 2′‐deoxyuridine 5′­monophosphate) for 72 h. These cells were then assessed by comet assay. ***P < 0.001. D–E) Levels of metabolites related to the TCA cycle in RFWD3 knockout cells and control cells. Data are presented as the mean ± SD with four replicates. ^*^ *P* < 0.05, ^**^ *P* < 0.01, ^***^ *P* < 0.001. F,G) 143B cells were transfected with the indicated siRNAs for 48 h. The levels of aspartic acid (F) and glutamic (G) acid were measured. Data are presented as the mean ± SD with three replicates. ^***^ *P* < 0.001, ^****^ *P* < 0.0001. H) 143B and U2OS cells expressing sgNC or sgRFWD3 were treated with or without 20mM aspartic acid for 48 h. These cells were then subjected to comet assay. I) 143B and U2OS cells expressing sgNC or sgRFWD3 were treated with or without NAD^+^ and measured with cellular aspartic acid level. ***P < 0.001, ****P < 0.0001.

Previous studies have established that NAD^+^ serves as a crucial substrate for poly (ADP‐ribose) polymerases (PARPs) during the DNA repair process, highlighting one of the mechanisms through which NAD^+^ contributes to cisplatin (DDP) resistance.^[^ ^19^ ^]^ However, our integration of metabolomics data has revealed a pronounced downregulation of purine, aspartate, and glutamate metabolic pathways in RFWD3 knockout osteosarcoma cells compared to the control group (**Figure** **6**A). Consequently, we hypothesized that RFWD3 also plays a regulatory role in nucleotide metabolism. Subsequent analysis of metabolites associated with nucleotide metabolism indicated a marked downregulation in RFWD3 knockout osteosarcoma cells compared to the control group (Figure 6B). A sufficient nucleotide pool is essential for effective DNA repair. We then demonstrated that supplementation with nucleotides could partially mitigate the impact of RFWD3 knockout on DDP sensitivity and DNA damage in 143B and U2OS cells (Figure 6C; Figure S4B–E, Supporting Information). Moreover, the knockdown of PHGDH partially reversed the effect of RFWD3 knockdown on DNA damage (Figure S4F,G, Supporting Information), indicating that RFWD3 regulated DNA repair partially through PHGDH. Collectively, these findings suggest that RFWD3 regulates nucleotide metabolism to sustain DNA repair.

NAD^+^ is a critical cofactor involved in the tricarboxylic acid (TCA) cycle.^[^ ^21^ ^]^ Consequently, we investigated the metabolite levels within the TCA cycle in RFWD3 knockout cells. The results demonstrated that RFWD3 knockout significantly decreased the levels of isocitric acid, oxoglutaric acid, asparagine, dephospho‐CoA, aspartic acid, and glutamic acid (Figure 6D,E). Notably, aspartic acid and glutamic acid were essential for nucleotide synthesis. Metabolite analysis confirmed a marked reduction in aspartic acid and glutamic acid levels in RFWD3 knockdown cells (Figure 6F,G). In addition, DDP treatment increased the cellular aspartic acid and glutamic acid levels (Figure S4H,I, Supporting Information). Exogenous aspartic acid supplementation partially mitigated the DNA damage induced by RFWD3 knockout in 143B and U2OS cells (Figure 6G). Moreover, exogenous NAD^+^ supplementation restored aspartic acid levels (Figure 6H). Collectively, these findings suggest that the RFWD3/PHGDH axis enhances nucleotide biosynthesis by augmenting cellular aspartic acid and glutamic acid production.

### 3.7 A Small‐Molecule Compound Enhances the Efficacy of Cisplatin in Osteosarcoma by Inhibiting the Interaction Between RFWD3 and PHGDH

**Figure 7:**
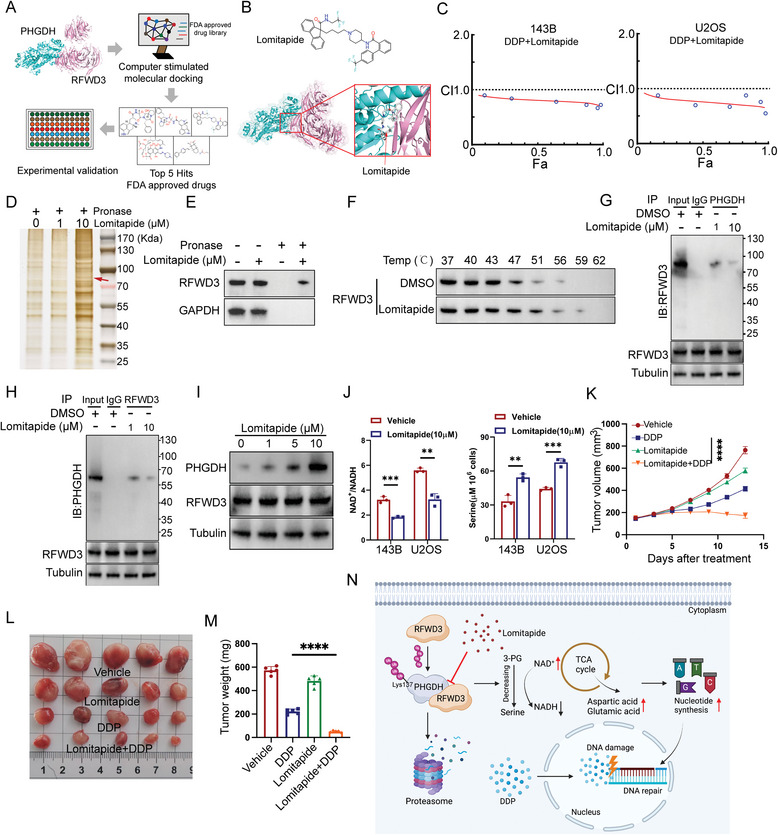
A small‐molecule compound enhances the efficacy of cisplatin in osteosarcoma by inhibiting the interaction between RFWD3 and PHGDH. A) A flowchart depicting the procedure for identifying small‐molecule inhibitors targeting the interaction between RFWD3 and PHGDH is presented. B) A diagram showing the structure of Lomitapide and the potential binding surface at the RFWD3‐PHGDH interaction. C) 143B and U2OS cells were treated with indicated drugs or drug combination for 48 h. These cells were then subjected to CCK‐8 assay and analyzed by Chou‐talalay method. D) The whole lysates of 143B cells were incubated with 0, 1.0, or 10 µm Lomitapide for 4 h and then digested using pronase. The lysates were then subjected to silver staining. E) The whole lysates of 143B cells were incubated with 10 µm Lomitapide for four hours and then digested using pronase. The lysates were then subjected to western blot analysis. F) The whole lysates of 143B cells were treated with DMSO or lomitapide (10 µm). After incubation at room temperature for 1 h, the cells in each group were heated at the indicated temperature for 5 min. The lysates were then subjected to western blot analysis. G,H) 143B cells were treated with DMSO or Lomitapide and subjected to co‐IP assay. I) 143B cells were treated with 0, 1, 5, and 10 µm Lomitapide for 48 hours. The whole lysates of these cells were collected for western blot analysis. J) 143B and U2OS cells were treated with 10 µm Lomitapide for 48 h and measured with the NAD+/NADH ratio and serine level. **P < 0.01, ***P < 0.001. K–M) 143B cells were subcutaneously injected into the nude mice. These mice were treated the indicated drugs or drug combination (DDP: 5 mg kg^−1^, twice a week, intraperitoneally, Lomitapide: 10 mg kg^−1^, every 2 days, intraperitoneally) starting when the tumor volume reached up to 100 mm^3^. Tumor growth curve (K), tumor images N), and tumor mass O) are shown. Data are presented as the mean ± SD with five replicates. ^****^ *P* < 0.0001. N. Working model illustrating how RFWD3 regulates DDP sensitivity.

As previously mentioned, the physical interaction between RFWD3 and PHGDH plays a critical role in mediating cisplatin resistance in osteosarcoma. To further investigate the binding sites and interaction mechanisms between RFWD3 and PHGDH, protein docking analysis was performed. The results showed that PHGDH (depicted in blue) and RFWD3 (depicted in orange) form hydrogen bonds at critical amino acid residues with a calculated binding energy of −19.3 kcal/mol. These findings suggest that PHGDH and RFWD3 form a stable protein complex, providing strong evidence for their potential interaction (**Figure** **7**A). To develop novel therapeutic strategies targeting the molecular mechanisms underlying cisplatin resistance mediated by the RFWD3‐PHGDH interaction, molecular docking simulations were performed using AutoDock Vina, a program designed to predict the binding mode of small molecules to protein targets.^[^ ^22^ ^]^ We first screened 2115 FDA‐approved small molecule compounds from the ZINC database,^[^ ^23^ ^]^ and finally identified five small molecules as candidate drugs: dihydroergotamine, ergotamine, lomitapide, rifampicin, and vorapaxar (Figure 7A). Further molecular dynamics analysis suggested that only lomitapide was able to bind at the RFWD3‐PHGDH interaction surface with the lowest binding energy (Figure 7B). Subsequent cisplatin sensitivity assays revealed that Lomitapide markedly enhanced the responsiveness of osteosarcoma cells to cisplatin therapy (Figure 7C). Lomitapide, an FDA‐approved lipid‐lowering drug, has been shown to prevent tumor cell growth and enhance therapeutic efficacy in multiple myeloma and glioma.^[^ ^24^, ^25^ ^]^ To confirm the interaction between lomitapide and RFWD3, we performed drug affinity responsive target stability (DARTS) and cell thermal shift assays (CETSA). These experiments demonstrated that lomitapide protected RFWD3 from pronase degradation and significantly enhanced its thermal stability (Figure 7D–F). Furthermore, co‐immunoprecipitation (Co‐IP) experiments validated that lomitapide effectively inhibited the protein‐protein interaction between RFWD3 and PHGDH (Figure 7G,H). Cellular experiments further confirmed that lomitapide treatment upregulated PHGDH protein levels, decreased cellular NAD+ levels, and increased concentrations of L‐serine (Figure 7I,J). To evaluate the therapeutic potential of lomitapide in osteosarcoma, we utilized a subcutaneous 143B cell mouse model to assess the efficacy of lomitapide combined with cisplatin. The results showed that the combination therapy significantly suppressed tumor growth compared to treatment with either agent alone (Figure 7K,L). In summary, lomitapide disrupts the interaction between RFWD3 and PHGDH, thereby enhancing cisplatin sensitivity in osteosarcoma. The combination of lomitapide and cisplatin represents a promising therapeutic strategy for osteosarcoma with significant potential for clinical application.

## 4 Discussion

The development of chemoresistance represents a significant impediment to effective osteosarcoma treatment and is a primary contributor to its poor prognosis. In the present study, we performed a genome‐wide CRISPR screen to identify the principal regulators of cisplatin resistance in osteosarcoma. Our findings confirm that RFWD3 functions as a critical mediator of DDP resistance. To date, no studies have elucidated the role of RFWD3 in chemoresistance. Further analysis revealed that RFWD3 modulates the nucleotide metabolism. Specifically, RFWD3 facilitates the ubiquitination and subsequent degradation of PHGDH, thereby inhibiting serine synthesis and reducing NAD^+^ consumption. The resultant surplus NAD^+^ enhances the TCA cycle, leading to increased production of aspartic acid and glutamic acid, which are essential for de novo nucleotide biosynthesis. Overall, our study elucidates the mechanistic underpinnings of RFWD3 regulation in DDP resistance and suggests that targeting RFWD3 and its associated metabolic pathways represents a promising strategy for osteosarcoma treatment.

DDP‐resistant cells exhibit a heightened activation of DNA repair pathways to mitigate the deleterious effects of DNA damage.^[^ ^26^ ^]^ Our previous studies have demonstrated that disturbance of the DNA repair pathway could sensitize osteosarcoma to chemotherapy.^[^ ^2^, ^27^ ^]^ RFWD3 is involved in multiple DNA repair processes, including RPA‐mediated DNA damage repair and the regulation of p53 stability.^[^ ^9^, ^28^ ^]^ However, our current study reveals that, in addition to its direct role in DNA repair, RFWD3 also modulates nucleotide metabolism in osteosarcoma. Enhanced nucleotide metabolism has been shown to facilitate DNA repair processes.^[^ ^6^ ^]^ Our observations indicate that DDP‐resistant cells reduce carbon flux through serine biosynthesis to spare NAD^+^. NAD^+^ is converted to poly ADP‐ribose under the catalysis of PARP, which participates in the DNA repair response.^[^ ^29^ ^]^ Previous studies have reported that some platinum‐resistant tumors downregulate PHGDH expression as part of metabolic reprogramming.^[^ ^19^ ^]^ The resistant cells rely on exogenous serine to sustain proliferation, while the expression of PHGDH, the key regulator of endogenous serine synthesis, is downregulated as part of a metabolic reprogramming associated with resistance. In line with this, we also demonstrated that the downregulation of PHGDH in resistant cells facilitates a shift toward an NAD+‐regenerating phenotype. NAD^+^ is involved in nucleotide metabolism through its regulation of the TCA cycle.^[^ ^30^ ^]^ We also found that the TCA cycle was suppressed in RFWD3 knockout cells and demonstrated that the level of aspartic acid and glutamic acid contributes to DDP resistance. These findings provide novel insights into the role of nucleotide metabolism in the chemoresistance of osteosarcoma, revealing unexpected vulnerabilities that can be therapeutically targeted.

Metabolic deregulation is an important feature of tumor biology and presents an effective target for cancer treatment.^[^ ^31^, ^32^ ^]^ Over the past decades, researchers have extensively investigated the role of metabolic reprogramming in the progression of osteosarcoma.^[^ ^33^, ^34^, ^35^, ^36^ ^]^ Additionally, studies have revealed that changes in metabolic processes can contribute to the development of chemoresistance of osteosarcoma.^[^ ^37^, ^38^ ^]^ However, these studies have not elucidated the detailed mechanisms by which these metabolic changes are regulated in osteosarcoma. In our study, we identified RFWD3 as a crucial E3 ubiquitin ligase for the metabolic enzyme PHGDH, ubiquitinating PHGDH at the Lys137 residue. This modification impacts PHGDH's stability, thereby influencing metabolic pathways critical for osteosarcoma chemoresistance. Previous research has confirmed that PHGDH can also be ubiquitinated by Parkin, another E3 ubiquitin ligase, at the Lys330 residue.^[^ ^39^ ^]^ Therefore, our findings suggest that the regulation of PHGDH by multiple ubiquitin ligases may be a significant factor in metabolic reprogramming and chemoresistance in osteosarcoma.

Lomitapide, an FDA‐approved lipid‐lowering drug, is primarily used to treat homozygous familial hypercholesterolemia. Previous studies have shown that lomitapide targets PARP14 for the treatment of multiple myeloma, and Song MM et al. identified lomitapide as an effective drug for glioma therapy through high‐throughput screening.^[^ ^24^, ^25^ ^]^ However, its role in osteosarcoma remains unknown. Through virtual screening and experimental validation, our data demonstrate that lomitapide is a potent inhibitor targeting RFWD3, effectively disrupting the interaction between RFWD3 and PHGDH. As a result, lomitapide enhanced the therapeutic sensitivity of osteosarcoma to cisplatin. The combination of lomitapide and cisplatin represents a promising therapeutic strategy for osteosarcoma. However, further clinical studies are needed to investigate the pharmacokinetics and pharmacodynamics of lomitapide, as well as to assess the drug combination effects and combination index of lomitapide and cisplatin using alternative models, such as tumor organoids, to support its clinical translation.

In summary, this study identifies the RFWD3/PHGDH axis as a pivotal regulator of DDP resistance in osteosarcoma. Mechanistically, RFWD3 ubiquitinates PHGDH at Lys137, promoting its degradation. The downregulation of PHGDH conserves NAD^+^, thereby enhancing the TCA cycle and promoting de novo nucleotide biosynthesis, which in turn supports DNA repair and contributes to DDP resistance in osteosarcoma. These findings provide novel insights into the metabolic vulnerabilities associated with chemoresistance in osteosarcoma.

## Conflict of Interest

The authors declare no conflict of interest.

## Author Contributions

W.Z. and C.Y. contributed equally to this work. W.Z. performed conceptualization, data curation, formal analysis, validation, investigation, visualization, methodology, wrote the original draft and edited the final manuscript. C.Y. performed conceptualization, data curation, formal analysis, validation, investigation, visualization, and methodology. L.Q. performed investigation, methodology, and resources. Z.L. performed investigation, methodology, and resources. R.X. performed investigation and methodology. C.T. performed conceptualization, resources, data curation, supervision, acquired funding acquisition, project administration, wrote reviewed and edited the final manuscript. Z.L. performed conceptualization, resources, data curation, formal analysis, supervision, acquired funding acquisition, investigation, visualization, project administration, wrote reviewed and edited the final manuscript.

## Supporting information



Supporting Information

## Data Availability

The data that support the findings of this study are available from the corresponding author upon reasonable request.
