# Supplementary material for: RFWD3 Reprograms Nucleotide Metabolism Through PHGDH to Induce Chemoresistance In Osteosarcoma
Source: Adv Sci (Weinh). 2025 Feb 28;12(16):2410937. doi: 10.1002/advs.202410937 (PMC12021087; doi:10.1002/advs.202410937)
Supplement: Supplementary file 1 — Supporting Information [file ADVS-12-2410937-s001.docx]

**Supplementary materials**


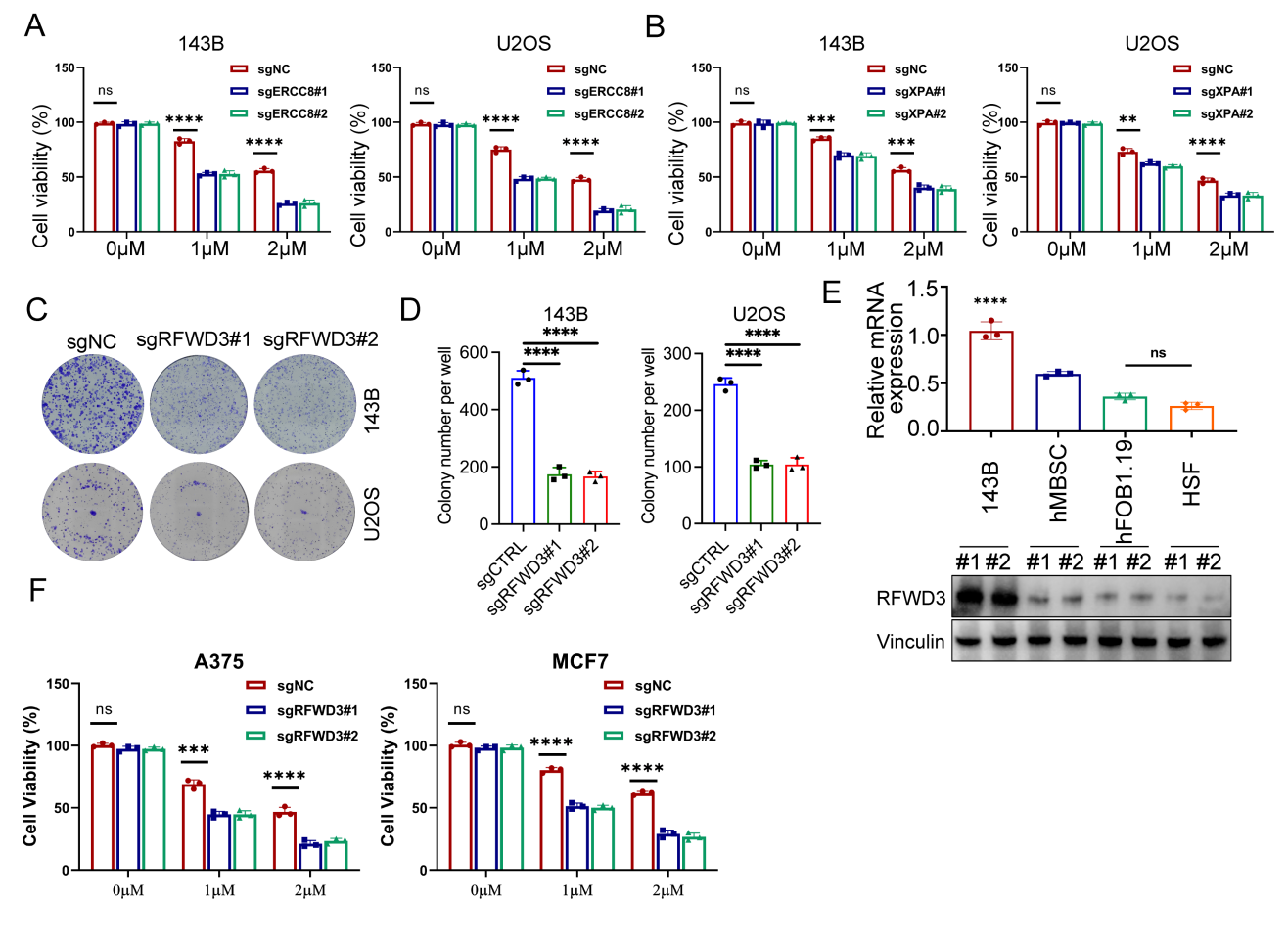


**Supplementary Figure 1**

1. B)143B and U2OS cells were transfected with indicated constructs sgRNAs for 48 hours. After puromycin selection, these cells were treated with 2 μM DDP and subjected to CCK-8 assay. (C-D) 143B and U2OS cells were transfected with indicated constructs for 48 h. After puromycin selection, these cells were subjected to colony formation assay. (E) mRNA and protein were extracted from 143B, hBMSC, hFOB1.19 and HSF cells for qPCR and western blot analysis. (F) A375 and MCF7 cells were transfected with indicated constructs sgRNAs for 48 h. After puromycin selection, these cells were treated with the indicated concentrations of DDP for 48 hours and subjected to CCK-8 assay.


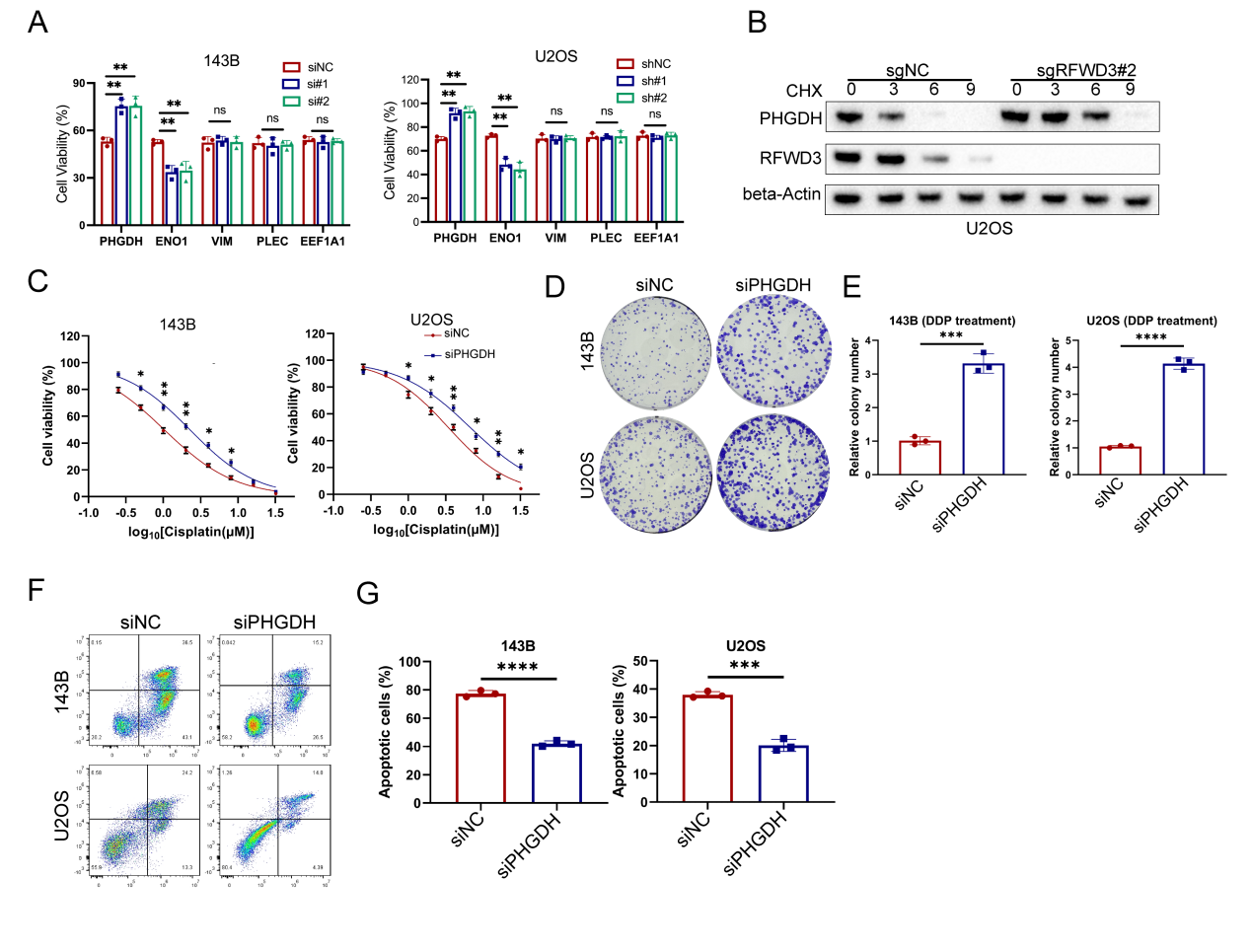


**Supplementary Figure 2**

1. 143B and U2OS cells were transfected with indicated siRNAs for 48 h. These cells were treated with 2 μM DDP for 48 hours and were subjected to CCK-8 assay. (B) U2OS cells were transfected with indicated constructs for 48h. After puromycin selection, these cells were treated with 100 mg/ml CHX for an indicted time gradient (0, 3, 6, 9 h) and were analyzed by Western blotting. (C) 143B and U2OS cells were transfected with indicated siRNAs for 48 hours. These cells were treated with different doses of DDP for 48 hours and were subjected to CCK-8 assay. (D-E) 143B and U2OS cells were transfected with indicated siRNAs for 48 h. These cells were then treated with 0.2μM DDP for 10 days and assessed with colony formation assay. (F-G) 143B and U2OS cells were transfected with indicated siRNAs for 48 h. These cells were treated with 2μM DDP for 48 hours and then assessed with apoptosis assay.


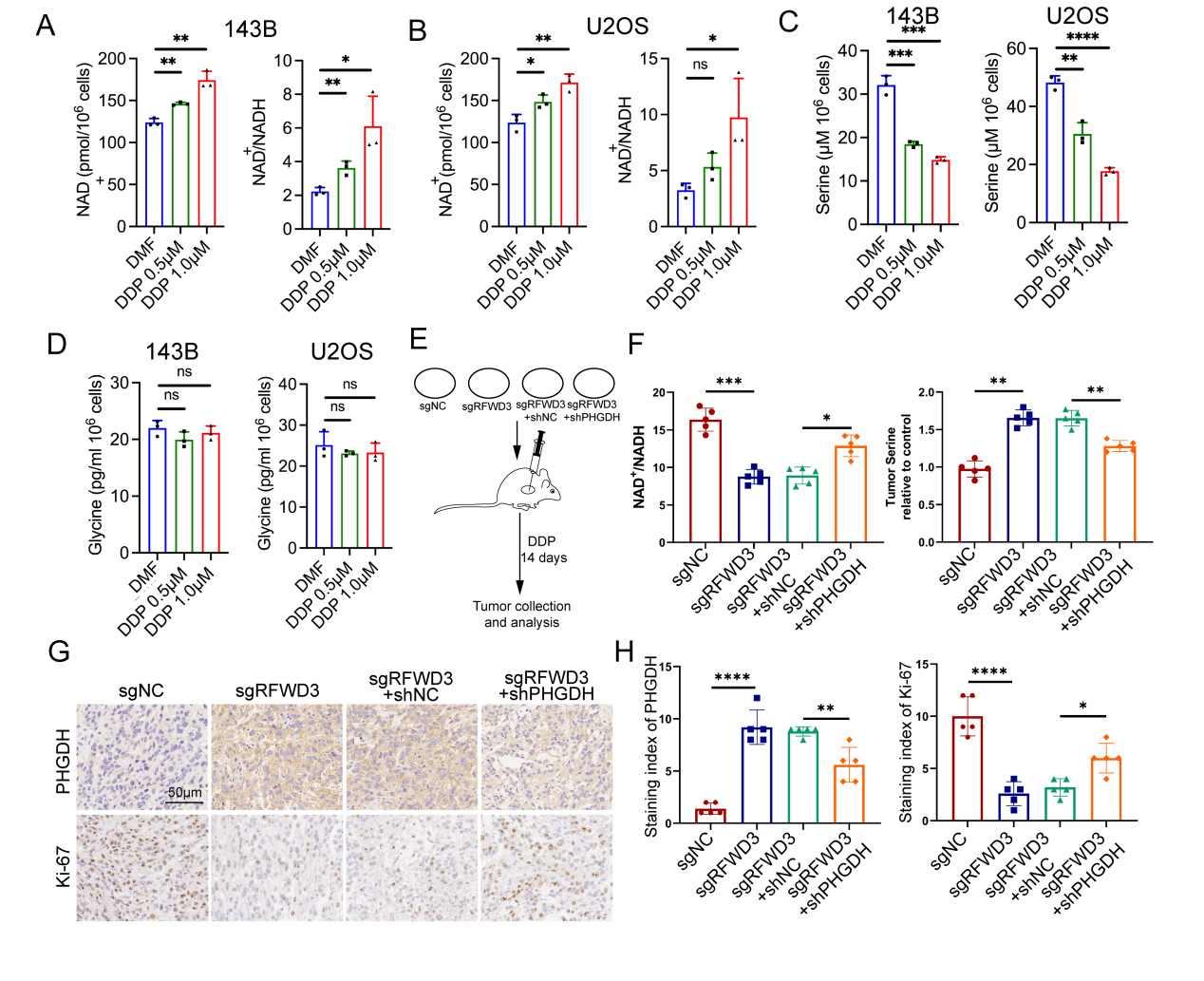


**Supplementary Figure 3**

1. 143B and U2OS cells were treated with indicated concentrations of DDP for 48 h and measured with the level of NAD+, NAD+/NADH ratio (A and B), serine (C), and glycine (D). (E-H) 143B cells were transfected with the indicated constructs for 48 h. After puromycin selection, these cells were subcutaneously injected into the nude mice. These mice were treated with 5 mg/kg DDP for 14 days, starting when the tumor reached 100 mm^3^. Tumors were collected for the analysis of NAD+/NADH ratio, serine level and PHGDH expression. Data are presented as the mean ± SD with five replicates. Ns non-significant, *P < 0.05, **P < 0.01, ***P < 0.001, ****P < 0.0001.


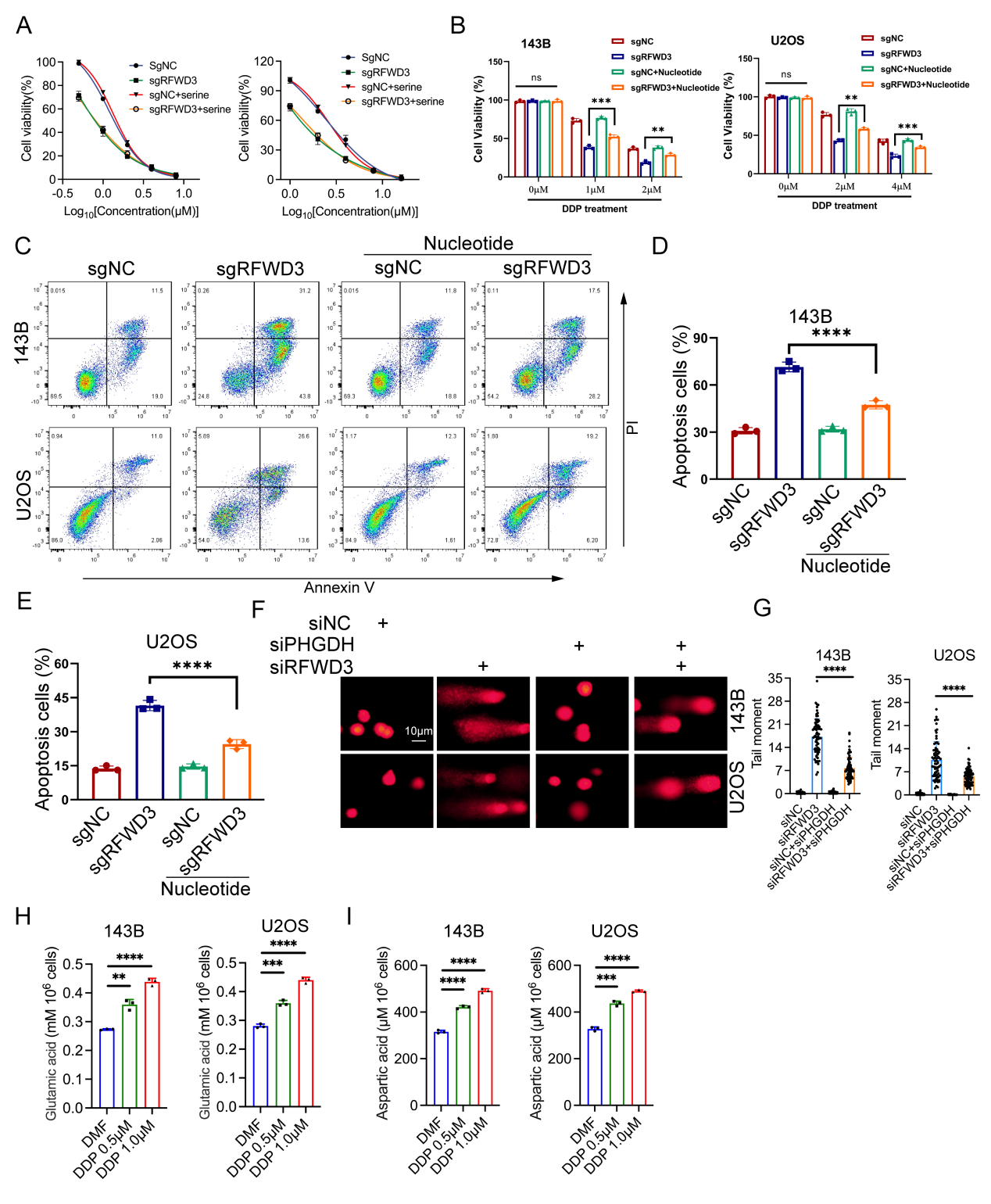


**Supplementary Figure 4**

1. 143B and U2OS cells expressing sgNC or sgRFWD3 were cultured in basic (DMEM: 42 mg/L, McCoy’s 5A: 26.3mg/L) or high L-serine (DMEM: 84 mg/L, McCoy’s 5A: 52.6 mg/L) culture medium for 72 hours under a range of DDP doses. These cells were then assessed by CCK-8 assay. (B) 143B and U2OS cells expressing sgNC or sgRFWD3 were treated with or without 12.5μM purines (2’-deoxyadenosine and 2’-deoxyguanosine) and 12.5μM pyrimidines (2’-deoxycytosine and 2’-deoxyuridine 5’­monophosphate) for 72 hours under the treatment of DDP. These cells were then assessed by CCK-8 assay. (C-E) 143B and U2OS cells expressing sgNC or sgRFWD3 were treated with or without 12.5μM purines (2’-deoxyadenosine and 2’-deoxyguanosine) and 12.5μM pyrimidines (2’-deoxycytosine and 2’-deoxyuridine 5’­monophosphate) for 72 hours under the treatment of 2 μM DDP. These cells were then assessed by Annexin V-FITC/PI assay. (F-G) 143B and U2OS cells were transfected with indicated siRNAs for 48 h. These cells were then assessed with comet assay. (H-I) 143B and U2OS cells were treated with indicated concentrations of DDP for 48 h and measured with the level of glutamic acid (H) and aspartic acid (I).


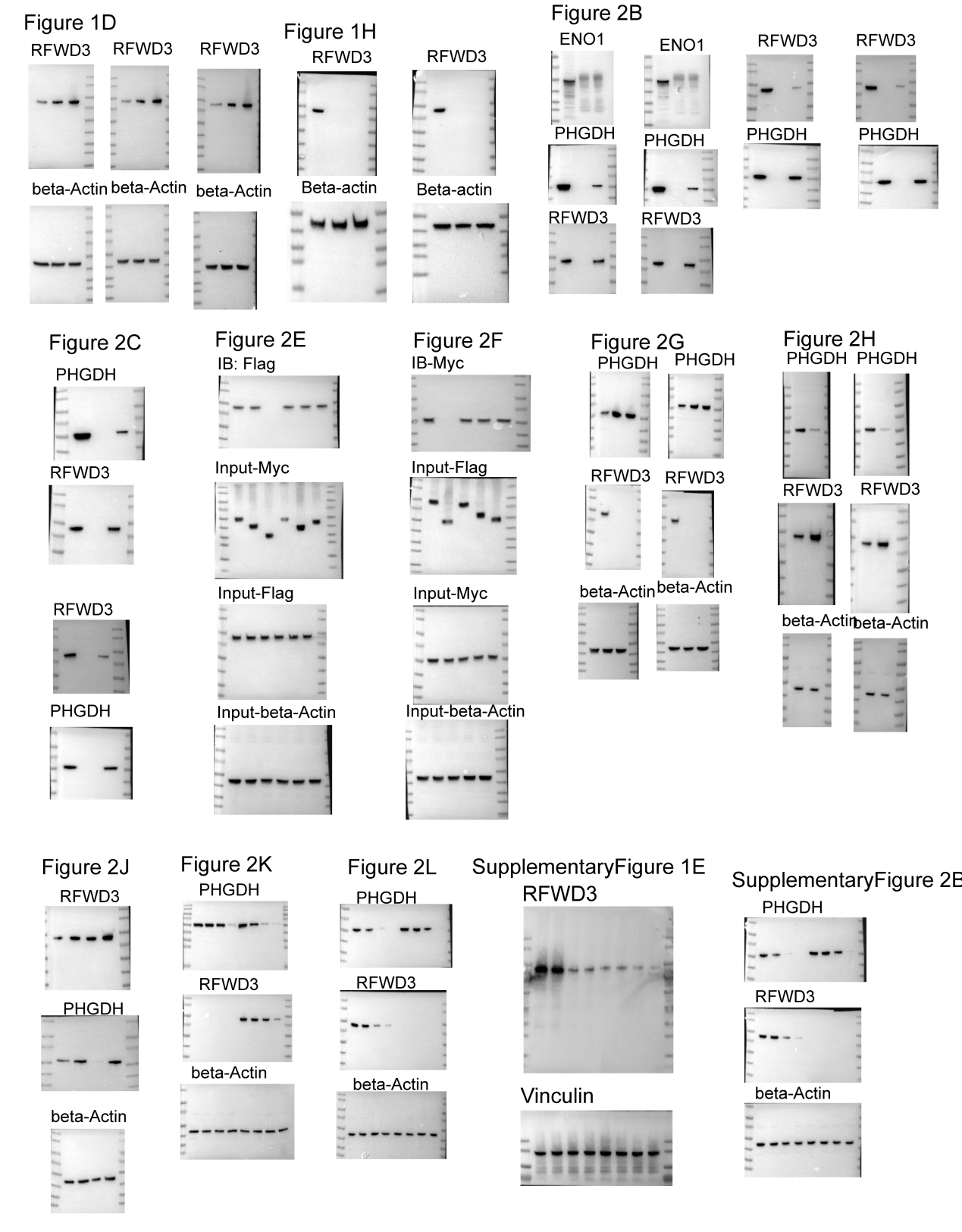


**Supplementary Figure 5** The full-blot data of WB


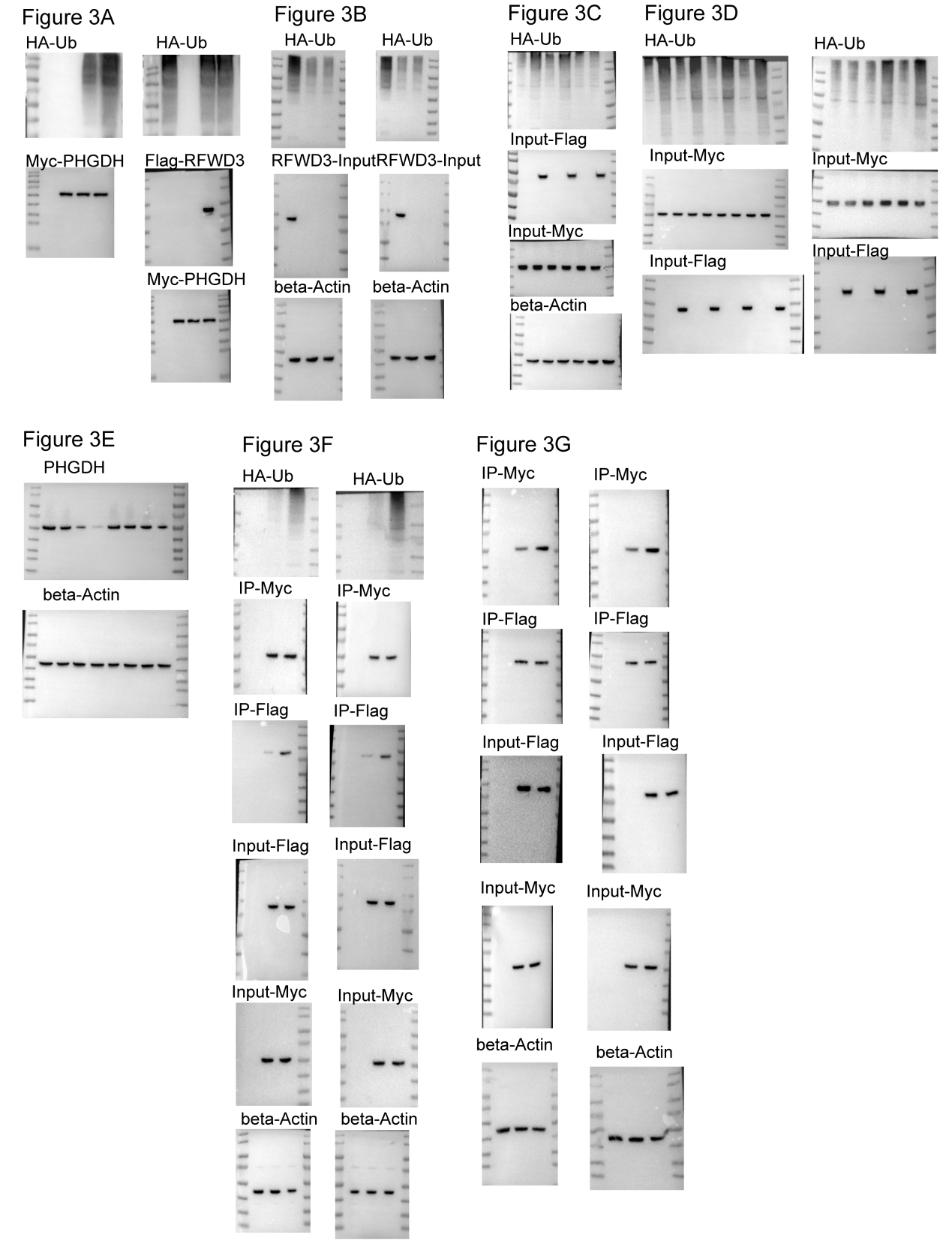


**Supplementary Figure 6** The full-blot data of WB


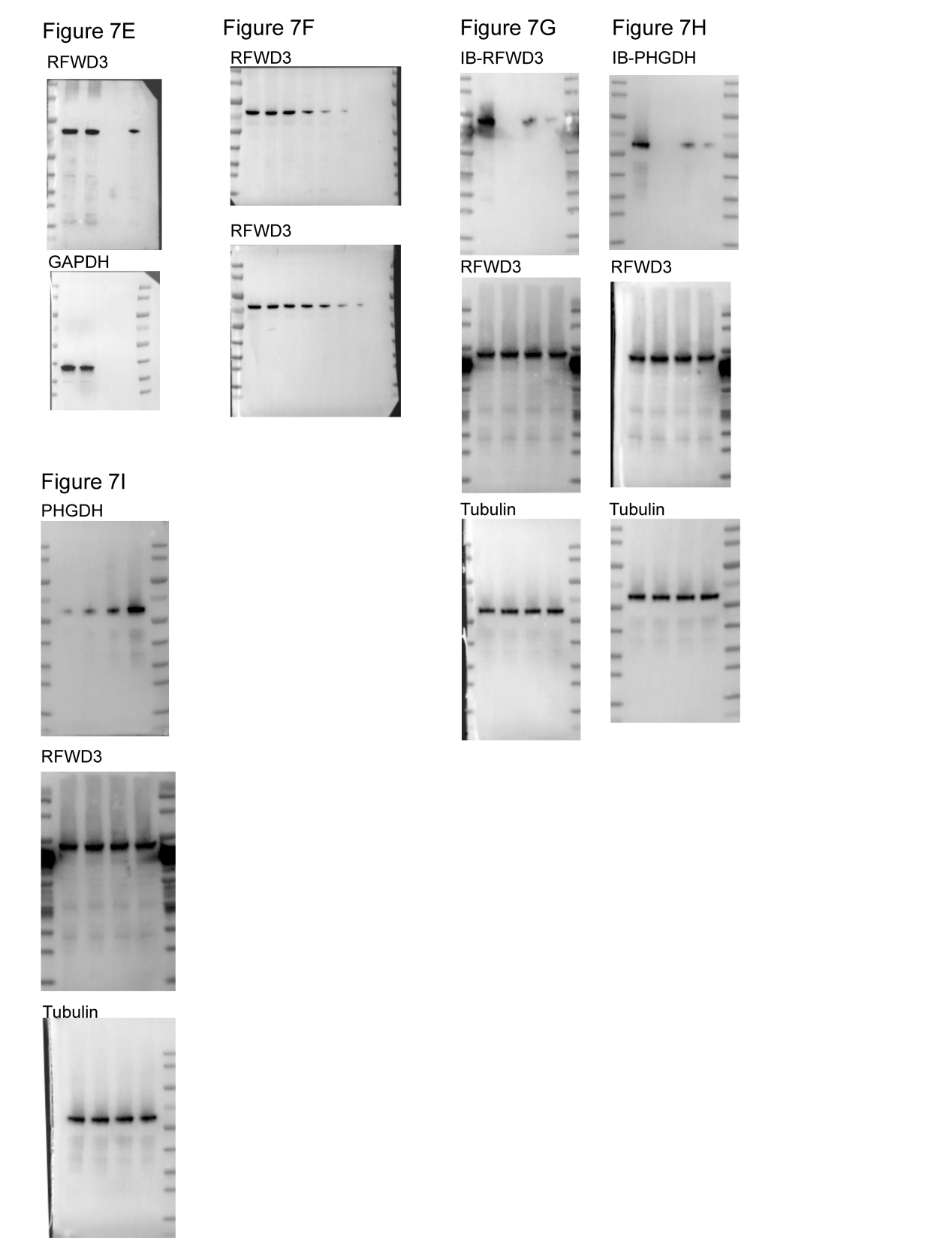


**Supplementary Figure 7** The full-blot data of WB

**Supplementary table 1** The sequences of primers, siRNAs, shRNA and sgRNAs used in this study.

| Name | Sequence |
| --- | --- |
| Primer-PHGDH-F | CTGCGGAAAGTGCTCATCAGT |
| Primer-PHGDH-R | TGGCAGAGCGAACAATAAGGC |
| Primer-ACTIN-F | CATGTACGTTGCTATCCAGGC |
| Primer-ACTIN-R | CTCCTTAATGTCACGCACGAT |
| siRNA-RFWD3#1-A | UAGCAAACAACAUCUAACC |
| siRNA-RFWD3#1-S | GGUUAGAUGUUGUUUGCUA |
| siRNA-PHGDH#1-S | UCAGCUCCUCUUUGCUAAG |
| siRNA-PHGDH#1-S | CUUAGCAAAGAGGAGCUGA |
| sgRNA-RFWD3#1 | TTCCATCAGCACACTTCGTA |
| sgRNA-RFWD3#2 | TCCTACCGACTGGATGACAC |
| sgRNA-ERCC8#1 | GCCAAGATATAGTCATAACG |
| sgRNA-ERCC8#2 | CAGTGGTATCCTCATGACAC |
| sgRNA-XPA#1 | TACCTGCAGTTATCACAAGT |
| sgRNA-XPA#2 | GCCCCAAAGATAATTGACAC |
| shRNA-ENO1-A | TGGTTGACTTTGAGCAGGAG |
| shRNA-ENO1-S | ATCTCACAGTGACCAACCCA |
| shRNA-VIM-A | UAACUCGCUAAAGCCUGUCUU |
| shRNA-VIM-S | GACAGGCUUUAGCGAGUUAUU |
| shRNA-PLEC-A | UUCCAUUGGAGACAUCUUCAG |
| shRNA-PLEC-S | GAAGAUGUCUCCAAUGGAAGC |
| shRNA-EEF1A1-A | UAGAACUUGUGAAACCAUCAA |
| shRNA-EEF1A1-S | GAUGGUUUCACAAGUUCUAUU |

**Supplementary table 2** The top negatively enriched genes of CRISPR screen

| gene | log2FC | p_value_neg | p_value_pos |
| --- | --- | --- | --- |
| ERCC8 | -1.914955557 | 0.995784981 | 0.008704304 |
| RFWD3 | -1.76403013 | 0.895955092 | 0.042656054 |
| XPA | -1.610016619 | 0.99833392 | 0.014341918 |
| USP7 | -1.455717065 | 0.996445552 | 0.024773917 |
| RAD18 | -1.429776269 | 0.998024384 | 0.005862292 |
| XRCC2 | -1.185556145 | 0.999730601 | 0.00105793 |
| POLH | -1.162551697 | 0.748247788 | 0.026250303 |
| C19orf40 | -1.128784158 | 0.8102222 | 0.034235576 |
| RAD51D | -1.043925847 | 0.988224289 | 0.008125945 |
| FANCA | -0.996995935 | 0.985598867 | 0.018645765 |
| ERCC6 | -0.977704206 | 0.999988713 | 0.000123431 |
| FANCG | -0.950998559 | 0.955576103 | 0.093247432 |
| SLX4 | -0.942813655 | 0.850675271 | 0.031120215 |
| ATG7 | -0.927342836 | 0.999041988 | 0.004480535 |
| BRIP1 | -0.9157408 | 0.879730209 | 0.003321923 |
| MCM9 | -0.913880184 | 0.997059774 | 0.023304499 |
| MAD2L2 | -0.899543354 | 0.996978663 | 0.023440948 |
| UVSSA | -0.849193812 | 0.954448035 | 0.02102806 |
| FANCF | -0.839467781 | 0.512767522 | 0.003954515 |
| DDX42 | -0.836478718 | 0.995044396 | 0.025446582 |
| FANCM | -0.835835131 | 0.537247507 | 0.200989335 |
| C17orf70 | -0.784665844 | 0.588667081 | 0.120753569 |
| REV3L | -0.78114712 | 0.998045575 | 0.011309799 |
| SLC25A37 | -0.767381839 | 0.990548935 | 0.024083186 |
| GTPBP6 | -0.76625988 | 0.997661517 | 0.003122754 |
